# Time-crystalline eigenstate order on a quantum processor

**DOI:** 10.1038/s41586-021-04257-w

**Published:** 2021-11-30

**Authors:** Xiao Mi, Matteo Ippoliti, Chris Quintana, Ami Greene, Zijun Chen, Jonathan Gross, Frank Arute, Kunal Arya, Juan Atalaya, Ryan Babbush, Joseph C. Bardin, Joao Basso, Andreas Bengtsson, Alexander Bilmes, Alexandre Bourassa, Leon Brill, Michael Broughton, Bob B. Buckley, David A. Buell, Brian Burkett, Nicholas Bushnell, Benjamin Chiaro, Roberto Collins, William Courtney, Dripto Debroy, Sean Demura, Alan R. Derk, Andrew Dunsworth, Daniel Eppens, Catherine Erickson, Edward Farhi, Austin G. Fowler, Brooks Foxen, Craig Gidney, Marissa Giustina, Matthew P. Harrigan, Sean D. Harrington, Jeremy Hilton, Alan Ho, Sabrina Hong, Trent Huang, Ashley Huff, William J. Huggins, L. B. Ioffe, Sergei V. Isakov, Justin Iveland, Evan Jeffrey, Zhang Jiang, Cody Jones, Dvir Kafri, Tanuj Khattar, Seon Kim, Alexei Kitaev, Paul V. Klimov, Alexander N. Korotkov, Fedor Kostritsa, David Landhuis, Pavel Laptev, Joonho Lee, Kenny Lee, Aditya Locharla, Erik Lucero, Orion Martin, Jarrod R. McClean, Trevor McCourt, Matt McEwen, Kevin C. Miao, Masoud Mohseni, Shirin Montazeri, Wojciech Mruczkiewicz, Ofer Naaman, Matthew Neeley, Charles Neill, Michael Newman, Murphy Yuezhen Niu, Thomas E. O’Brien, Alex Opremcak, Eric Ostby, Balint Pato, Andre Petukhov, Nicholas C. Rubin, Daniel Sank, Kevin J. Satzinger, Vladimir Shvarts, Yuan Su, Doug Strain, Marco Szalay, Matthew D. Trevithick, Benjamin Villalonga, Theodore White, Z. Jamie Yao, Ping Yeh, Juhwan Yoo, Adam Zalcman, Hartmut Neven, Sergio Boixo, Vadim Smelyanskiy, Anthony Megrant, Julian Kelly, Yu Chen, S. L. Sondhi, Roderich Moessner, Kostyantyn Kechedzhi, Vedika Khemani, Pedram Roushan

**Affiliations:** 1 Google Research, Mountain View, CA USA; 2 Department of Physics, Stanford University, Stanford, CA USA; 3 Department of Electrical and Computer Engineering, University of Massachusetts, Amherst, MA USA; 4 Pritzker School of Molecular Engineering, University of Chicago, Chicago, IL USA; 5 Department of Electrical and Computer Engineering, University of California, Riverside, CA USA; 6 Department of Chemistry, Columbia University, New York, NY USA; 7 Department of Physics, University of California, Santa Barbara, CA USA; 8 Department of Physics, Princeton University, Princeton, NJ USA; 9 Rudolf Peierls Centre for Theoretical Physics, University of Oxford, Oxford, UK; 10 Max-Planck-Institut für Physik komplexer Systeme, Dresden, Germany

**Keywords:** Phase transitions and critical phenomena, Quantum simulation, Quantum information, Statistical physics

## Abstract

Quantum many-body systems display rich phase structure in their low-temperature equilibrium states^1^. However, much of nature is not in thermal equilibrium. Remarkably, it was recently predicted that out-of-equilibrium systems can exhibit novel dynamical phases^2–8^ that may otherwise be forbidden by equilibrium thermodynamics, a paradigmatic example being the discrete time crystal (DTC)^7,9–15^. Concretely, dynamical phases can be defined in periodically driven many-body-localized (MBL) systems via the concept of eigenstate order^7,16,17^. In eigenstate-ordered MBL phases, the entire many-body spectrum exhibits quantum correlations and long-range order, with characteristic signatures in late-time dynamics from all initial states. It is, however, challenging to experimentally distinguish such stable phases from transient phenomena, or from regimes in which the dynamics of a few select states can mask typical behaviour. Here we implement tunable controlled-phase (CPHASE) gates on an array of superconducting qubits to experimentally observe an MBL-DTC and demonstrate its characteristic spatiotemporal response for generic initial states^7,9,10^. Our work employs a time-reversal protocol to quantify the impact of external decoherence, and leverages quantum typicality to circumvent the exponential cost of densely sampling the eigenspectrum. Furthermore, we locate the phase transition out of the DTC with an experimental finite-size analysis. These results establish a scalable approach to studying non-equilibrium phases of matter on quantum processors.

## Abstract (web summary)

A study establishes a scalable approach to engineer and characterize a many-body-localized discrete time crystal phase on a superconducting quantum processor.

## Main

**Fig. 1:**
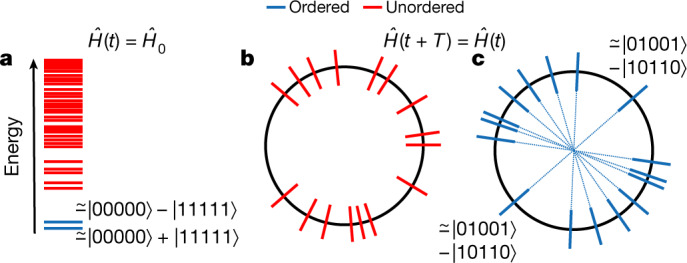
Order in eigenstates. **a**, Equilibrium phases are characterized by long-range order in low-energy eigenstates of time-independent Hamiltonians (for example, an Ising ferromagnet with a pair of degenerate ground states that resemble ‘Schrödinger cats’ of polarized states). **b**, Thermalizing Floquet systems typically have no ordered states in the spectrum. **c**, In MBL Floquet systems, every eigenstate can show order. In MBL-DTC, every eigenstate resembles a long-range ordered ‘Schrödinger cat’ of a random configuration of spins and its inversion, with even/odd superpositions split by π.

In an equilibrium setting, quantum phases of matter are classified by long-range order or broken symmetries in low-temperature states (Fig. 1a). The existence of ordered phases in periodically driven (Floquet) systems, on the other hand, is counterintuitive: as energy is not conserved, one expects thermalization to a featureless maximum-entropy state that is incompatible with quantum order. However, this heat death is averted in the presence of many-body localization, where strong disorder causes the emergence of an extensive number of local conservation laws that prevent thermalization^18–23^, making it possible to stabilize intrinsically dynamical phases^7^.

Dynamics in a Floquet system is governed by a unitary time evolution operator, whose eigenvalues lie on the unit circle. While the entire Floquet spectrum is featureless in a thermalizing phase (Fig. 1b), an MBL Floquet phase can have an order parameter associated with each eigenstate. As an example, in the spatiotemporally ordered MBL-DTC, the spectrum has a distinctive pattern of pairing between long-range ordered ‘Schrödinger cat’ eigenstates whose eigenvalues are separated by an angle π (refs. ^7,9,10^; Fig. 1c). This pairing manifests as a stable subharmonic response, wherein local observables show period-doubled oscillations that spontaneously break the discrete time translation symmetry of the drive for infinitely long times. The unique combination of spatial long-range order and time translation symmetry breaking in an isolated dissipation-free quantum many-body system is the hallmark of the MBL-DTC.

Experimentally observing a non-equilibrium phase such as the MBL-DTC is a challenge owing to limited programmability, coherence and size of noisy intermediate-scale quantum hardware. Subharmonic response, by itself, is not a unique attribute of the MBL-DTC; rather, it is a feature of many dynamical phenomena whose study has a rich history^24^ (also Ch. 8 in ref. ^12^). Most recently, interesting DTC-like dynamical signatures have been observed in a range of quantum platforms from trapped ions^25^ to nitrogen vacancy centres^26^ to NMR spins^27,28^. However, each of these platforms lacks one or more necessary conditions for stabilizing an MBL-DTC^12,29^, either owing to an absence of the requisite type of disorder^25,27^ or owing to the interactions being too long ranged^26–28^. The observed signatures, instead, have been shown to arise from slow thermalization^26,30^, effectively mean-field dynamics^28^, or prethermal dynamics from special initial states^12,29,31,32^, and are separated from the MBL-DTC by a spectral phase transition where eigenstate order disappears. Thus, despite the recent progress, observing an MBL-DTC remains an outstanding challenge^12,29^.

Here we perform the following necessary benchmarks for experimentally establishing an eigenstate-ordered non-equilibrium phase of matter: drive parameters are varied to demonstrate stability of the phase in an extended parameter region and across disorder realizations; the limitations of finite size and finite coherence time are addressed, respectively, by varying system size and verifying that any decay of the subharmonic response is consistent with purely extrinsic decoherence assessed in an independent experiment; the existence of spatiotemporal order across the entire spectrum is established. The flexibility of our quantum processor, combined with the scalable experimental protocols devised in the following, allows us to fulfil these criteria and observe an MBL-DTC.

**Fig. 2:**
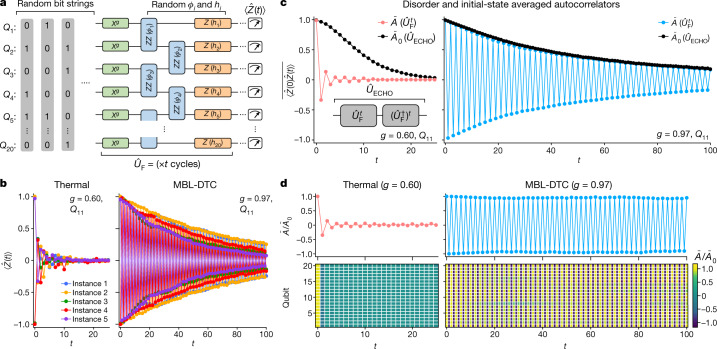
Observing an MBL-DTC. **a**, The experimental circuit composed of $t$ identical cycles of the unitary ${\hat{U}}_{{\rm{F}}}$. The local polarization of each qubit, ⟨$\hat{Z}(t)$⟩, is measured at the end. In the following panels, we investigate a number of disorder instances each with a different random bit-string initial state. **b**, Experimental values of ⟨$\hat{Z}(t)$⟩ measured at *Q*_11_. Data are shown for five representative circuit instances deep in the thermal (*g* = 0.60; left) and MBL-DTC (*g* = 0.97; right) phases. **c**, Autocorrelator $\bar{A}=\overline{\langle \hat{Z}(0)\hat{Z}(t)}\rangle$ at *Q*_11_, obtained from averaging the results of 36 circuit instances. For the same circuit instances, the average autocorrelator at the output of ${\hat{U}}_{{\rm{ECHO}}}={({\hat{U}}_{{\rm{F}}}^{\dagger })}^{t}{\hat{U}}_{{\rm{F}}}^{t}$ is also measured and its square root, ${\bar{A}}_{0}$, is shown alongside $\bar{A}$ for comparison. The left (right) panels correspond to *g* = 0.60 (0.97). **d**, Top panels: the ratio $\bar{A}/{\bar{A}}_{0}$ obtained from **c**. Bottom panels: $\bar{A}/{\bar{A}}_{0}$ as a function of $t$ and qubit location. The left (right) panels correspond to *g* = 0.60 (0.97) .

The experiment is conducted on an open-ended, linear chain of $L=20$ superconducting transmon qubits (${Q}_{1}$ to ${Q}_{20}$) that are isolated from a two-dimensional grid. We drive the qubits via a time-periodic (Floquet) circuit ${\hat{U}}_{{\rm{F}}}^{t}$ with $t$ identical cycles (Fig. 2a) of ${\hat{U}}_{{\rm{F}}}$:

1 $${\hat{U}}_{{\rm{F}}}=\mathop{\underbrace{{{\rm{e}}}^{-\frac{i}{2}\sum _{i}{h}_{i}{\hat{Z}}_{i}}}}\limits_{{\rm{longitudinal}}\,{\rm{fields}}}\,\mathop{\underbrace{{{\rm{e}}}^{-\frac{i}{4}\sum _{i}{\varphi }_{i}{\hat{Z}}_{i}{\hat{Z}}_{i+1}}}}\limits_{{\rm{Ising}}\,{\rm{interaction}}}\,\mathop{\underbrace{{{\rm{e}}}^{-\frac{i}{2}{\rm{\pi }}g\sum _{i}{\hat{X}}_{i}}}}\limits_{x\,{\rm{rotation}}\,{\rm{by}}\,{\rm{\pi }}g}$$

where ${\hat{X}}_{i}$ and ${\hat{Z}}_{i}$ are Pauli operators. Each angle ${\varphi }_{i}$ $({h}_{i})$ is sampled randomly from $[-1.5{\rm{\pi }},-0.5{\rm{\pi }}]$ $([-{\rm{\pi }},{\rm{\pi }}])$ for every realization of the circuit. Overall, ${\hat{U}}_{{\rm{F}}}$ implements an interacting Ising model that is periodically ‘kicked’ by a transverse pulse that rotates all qubits by ${\rm{\pi }}g$ about the $x$ axis. In this work, $g$ is tuned within the range $[0.5,\,1.0]$to explore the DTC phase and its transition into a thermal phase. At $g=1$, the model implements a ${\rm{\pi }}$ pulse that exactly flips all qubits (in the $z$ basis) and returns them to the initial state over two periods. A key signature of the DTC is the presence of robust period doubling, (that is, extending over a finite extent in parameter space, even as $g$ is tuned away from $1$, and for all initial states). Strong Ising interactions, which produce long-range spatial order, are essential for this robustness^7,10^. This is in contrast to a system of decoupled qubits $(\varphi =0)$ that rotate by a continuously varying angle ${\rm{\pi }}g$ every period instead of being locked at period doubling. Prior theoretical work^29^ has shown that model (1) is expect the range $g > {g}_{{\rm{c}}}$, and transition to a thermal phase at a critical value ${g}_{{\rm{c}}}\approx 0.84$.

Achieving MBL in this model for $g\sim 1$ requires disorder in the two-qubit interaction, ${\varphi }_{i}$, which is even under Ising symmetry^12,29^, ${{\rm{\pi }}}_{i}{\hat{X}}_{i}$, a condition that was not met by some past DTC experiments^25,27^. Ising-odd terms (that is, ${h}_{i}$) are approximately dynamically decoupled by the $x$ pulses over two periods, thereby lowering their effective disorder strength and hindering localization (in the absence of independent disorder in the ${\varphi }_{i}$); see Appendix A in ref. ^29^. Utilizing continuously tunable CPHASE gates, described further in the Supplementary Information, allows us to engineer strong disorder in ${\varphi }_{i}$ to fulfil this key requirement. Recently, a complementary approach to MBL-DTC using nuclear spins in diamond has also come into fruition^33^.

We first measure the hallmark of an MBL-DTC: the persistent oscillation of local qubit polarizations ⟨ $\hat{Z}(t)$⟩at a period twice that of ${\hat{U}}_{{\rm{F}}}$, irrespective of the initial state^7,9,12,29^. This subharmonic response is probed using a collection of random bit-string states (for example, $|\mathrm{01011...}$⟩, where $0$ (1) denotes a single-qubit ground (excited) state in the $z$ basis). For each bit-string state, we generate a random instance of ${\hat{U}}_{{\rm{F}}}$, and then measure ⟨$\hat{Z}(t)$⟩ every cycle. Figure 2b shows ⟨$\hat{Z}(t)$⟩in a few different instances for a qubit near the centre of the chain, ${Q}_{11}$, measured with $g=0.60$ and $g=0.97$. The former is deep in the thermal phase, and indeed we observe rapid decay of ⟨$\hat{Z}(t)$⟩ towards 0 within 10 cycles for each instance. In contrast, for $g=0.97$, ⟨$\hat{Z}(t)$⟩ shows large period-doubled oscillations persisting to over 100 cycles, suggestive of an MBL-DTC phase. The disorder-averaged autocorrelator, $\bar{A}=\overline{\langle \hat{Z}(0)\hat{Z}(t)\rangle }$, shows similar features (Fig. 2c).

We note that the data for $g=0.97$ are modulated by a gradually decaying envelope, which may arise from either external decoherence or slow internal thermalization^26,30^. To establish DTC, additional measurements are needed to quantify the impact of decoherence. This is achieved via an ‘echo’ circuit ${\hat{U}}_{{\rm{ECHO}}}={({\hat{U}}_{{\rm{F}}}^{\dagger })}^{t}{\hat{U}}_{{\rm{F}}}^{t}$ that reverses the time evolution after *t* steps (see Supplementary Information). Deviations of ${\hat{U}}_{{\rm{ECHO}}}$ from the identity operation are purely due to decoherence, and can be quantified via decay of the autocorrelator ${A}_{0}\equiv {(\langle \hat{Z}{\hat{U}}_{{\rm{ECHO}}}^{\dagger }\hat{Z}{\hat{U}}_{{\rm{ECHO}}}\rangle )}^{1/2}$ (the square root accounts for the fact that ${\hat{U}}_{{\rm{ECHO}}}$ acts twice as long as ${\hat{U}}_{{\rm{F}}}^{t}$). Time-reversal techniques were also recently used in an investigation of DTC in NMR systems^27^ and the study of out-of-time-ordered commutators^34^.

Comparison between the disorder-averaged ${\bar{A}}_{0}$ and $\bar{A}$ reveals qualitatively different behaviours in the two phases (Fig. 2c). In the thermal phase $g=0.60$, $\bar{A}$ approaches 0 much more quickly than ${\bar{A}}_{0}$ does, indicating that the observed decay of $\bar{A}$ is mostly induced by intrinsic thermalization. In the MBL-DTC phase $g=0.97$, ${\bar{A}}_{0}$ nearly coincides with the envelope of $\bar{A}$, suggesting that decay of the latter is primarily induced by decoherence. We also find, consistent with theoretical models (see Supplementary Section IV), that the reference signal ${\bar{A}}_{0}$ may be used to normalize $\bar{A}$ and reveal its ideal behaviour: $\bar{A}/{\bar{A}}_{0}$, shown in the upper panels of Fig. 2d, decays rapidly for $g=0.60$ but retains near-maximal amplitudes for $g=0.97$. Similar contrast between the two phases is seen in the error-mitigated autocorrelators $\bar{A}/{\bar{A}}_{0}$ for all qubits (bottom panels of Fig. 2d). The observation of a stable noise-corrected subharmonic response is suggestive of an MBL-DTC phase.

We now demonstrate the insensitivity of the subharmonic response to the choice of initial states, a necessary consequence of eigenstate order. In contrast, various prethermal mechanisms in driven systems predict strong dependence of the thermalization rate on the initial state (for example, through its quantum numbers^27,32^ or its energy under an effective time-independent Hamiltonian ${\hat{H}}_{{\rm{eff}}}$ (refs. ^31,35,36^) that approximately governs the dynamics for small system sizes and/or finite times). To elucidate this aspect of the MBL-DTC phase, we measure in detail the distribution of autocorrelator values over initial bit-string states.

**Fig. 3:**
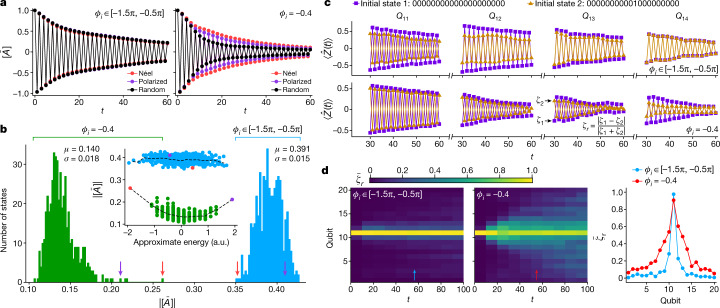
Distinguishing MBL-DTC from prethermal phenomena. **a**, Site- and disorder-averaged autocorrelators $[\bar{A}]$ measured with $g=0.94$. In the left panel (MBL-DTC), each dataset is averaged over 24 disorder instances of ${\varphi }_{i}$ and ${h}_{i}$, with the initial state fixed at one of the following: Néel, $|01{\rangle }^{\otimes 10}$; polarized, $|0{\rangle }^{\otimes 20}$; random, $|00111000010011001111$⟩. In the right panel (prethermal), the same values of ${h}_{i}$ and initial states are used but ${\varphi }_{i}=-\,0.4$. **b**, Histograms of$|[\bar{A}]|$, from 500 random bit-string initial states, averaged over cycles 30 and 31 and the same disorder instances as in **a**. The standard deviation (mean) of$|[\bar{A}]|$, $\sigma$ (*μ*), is also listed. Location of the polarized (Néel) state is indicated by a purple (red) arrow. Inset: same collection of$|[\bar{A}]|$plotted over the energies of the bit-string states, calculated from the effective Hamiltonian ${\hat{H}}_{{\rm{eff}}}$ approximating the drive (see text). Dashed lines show averaged values within energy windows separated by 0.2. **c**, ⟨$\hat{Z}(t)$⟩ for two bit-string initial states that differ only at ${Q}_{11}$. Top panel shows a single circuit instance with disordered ${\varphi }_{i}$ and bottom panel shows an instance with uniform ${\varphi }_{i}=-\,0.4$. **d**, Left and middle panels: relative difference between the two signals ${\bar{\zeta }}_{{\rm{r}}}$ as a function of *t* and qubit location, averaged over time windows of 10 cycles and over 64 disorder instances for ${\hat{U}}_{{\rm{F}}}$ and 81 instances for ${\hat{U}}_{{\rm{F}}\text{'}}$. Right panel: qubit dependence of ${\bar{\zeta }}_{{\rm{r}}}$, averaged from $t=51$ to $t=60$.

We begin by examining the position- and disorder-averaged autocorrelator $[\bar{A}]$ over three representative bit-string initial states, shown in the left panel of Fig. 3a. The square brackets indicate averaging over qubits in the chain. The three time traces are nearly indistinguishable. This behaviour is in clear contrast with a model without eigenstate order, implemented by a family of drives ${\hat{U}}_{{\rm{F}}}^{\text{'}}$ where the ${\varphi }_{i}$ angles are set to a uniform value, ${\varphi }_{i}=-\,0.4$. Note that this value of ${\varphi }_{i}=-\,0.4$ is chosen to be small enough that a leading-order high-frequency Floquet–Magnus expansion to obtain ${\hat{H}}_{{\rm{eff}}}$ is a reasonable approximation (see Supplementary Information). Without disorder in the ${\varphi }_{i}$, the drive ${\hat{U}}_{{\rm{F}}}^{\text{'}}$ is not asymptotically localized but exhibits prethermal DTC-like behaviour (see Methods). Here, $[\bar{A}]$ for ${\hat{U}}_{{\rm{F}}}^{\text{'}}$ (disorder averaged over random ${h}_{i}$ alone), shown in the right panel of Fig. 3a, reveals markedly different decay rates for the three states. The random bit-string state, in particular, decays faster than the polarized or Néel states.

A more comprehensive analysis is based on sampling the absolute values of $[\bar{A}]$ for 500 random initial bit-string states (Fig. 3b). For the MBL-DTC ${\hat{U}}_{{\rm{F}}}$, the histogram is symmetrical with a mean $\mu =0.391$. Here the non-zero standard deviation $\sigma$ probably arises from finite experimental accuracy and number of disorder instances, as analysis in the Supplementary Information shows that $[\bar{A}]$is independent of the initial state. In contrast, the ${\hat{U}}_{{\rm{F}}}^{\text{'}}$ model has a significantly lower mean $\mu =0.140$. Moreover, the histogram is asymmetrical, with outliers at high $[\bar{A}]$ including the polarized and Néel states (51% and 88% higher than the mean, respectively). These two states are special because they are low-temperature states that sit near the edge of the spectrum of ${\hat{H}}_{{\rm{eff}}}$ (see Supplementary Information). Plotting the autocorrelator $[\bar{A}]$ against the energy of each bit string under ${\hat{H}}_{{\rm{eff}}}$, in the inset of Fig. 3b, reveals a clear correlation. No such correlation is present in the MBL model.

Independent confirmation of MBL as the mechanism underlying the stability of DTC is achieved by characterizing the propagation of correlations. In MBL dynamics, local perturbations spread at most logarithmically in time^20^, as opposed to algebraic $(\sim {t}^{\alpha })$ spreading in thermalizing dynamics. We prepare two initial bit-string states differing by only a single bit flip at ${Q}_{11}$ and measure ⟨$\hat{Z}(t)$⟩ for each site in both states (Fig. 3c). It can be seen that the difference in the two signals, ${\zeta }_{1}$ and ${\zeta }_{2}$, decays rapidly with the distance from ${Q}_{11}$ for disordered ${\varphi }_{i}$ and becomes undetectable at ${Q}_{14}$. On the other hand, for uniform ${\varphi }_{i}=-0.4$, ${\zeta }_{1}$ and ${\zeta }_{2}$ have a much more pronounced difference that remains significant at ${Q}_{14}$. This difference is further elucidated by the ratio ${\zeta }_{{\rm{r}}}=|{\zeta }_{1}-{\zeta }_{2}|/(|{\zeta }_{1}|+|{\zeta }_{2}|)$, shown in Fig. 3d. Physically, ${\zeta }_{{\rm{r}}}$ corresponds to the relative change in local polarization as a result of the bit flip, and is inherently robust against qubit decoherence (see Supplementary Information). We observe that up to $t=100$, ${\zeta }_{{\rm{r}}}$ remains sharply peaked around the initial perturbation $({Q}_{11})$ for disordered ${\varphi }_{i}$. In contrast, a propagating light cone is visible for ${\varphi }_{i}=-\,0.4$, with the perturbation reaching all qubits across the chain as $t$ increases. The spatial profiles of ${\zeta }_{{\rm{r}}}$ at $t=51$ to $t=60$ (right panel of Fig. 3d) show that ${\zeta }_{{\rm{r}}}$ is much sharper for disordered ${\varphi }_{i}$. This slow propagation provides another experimental diagnostic in support of MBL.

**Fig. 4:**
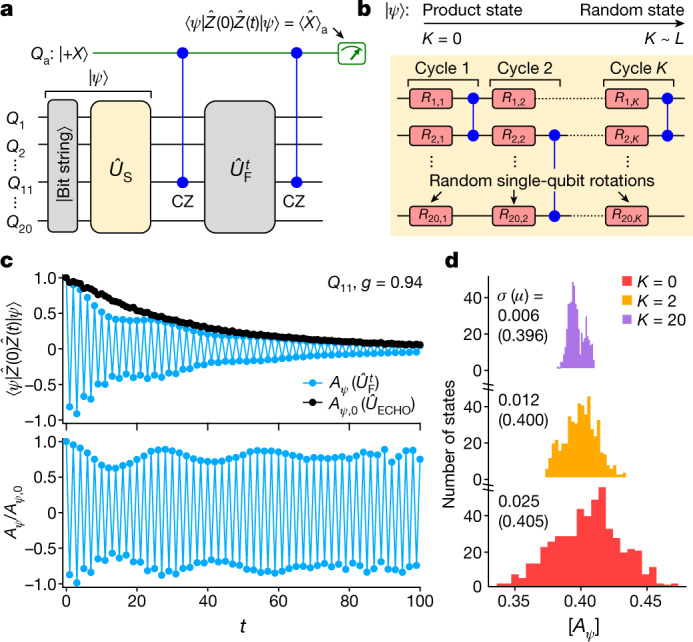
Probing average spectral response via quantum typicality. **a**, Scheme for measuring the autocorrelator, ${A}_{\psi }=\langle \psi |\hat{Z}(0)\hat{Z}(t)|\psi \rangle$, on ${Q}_{11}$, of a scrambled quantum state $|\psi$⟩. $|\psi$⟩ is created by scrambling a bit-string state with a circuit ${\hat{U}}_{{\rm{S}}}$. The *x*-axis projection of an ancilla qubit ${Q}_{{\rm{a}}}$, ${\langle \hat{X}\rangle }_{{\rm{a}}}$, is measured at the end. **b**, ${\hat{U}}_{{\rm{S}}}$ contains $K$ layers of controlled-Z (CZ) gates interleaved with random single-qubit rotations, ${R}_{i,k}$, around a random axis along the equatorial plane of the Bloch sphere by an angle $\in [0.4\pi ,0.6\pi ]$. **c**, Upper panel: ${A}_{\psi }$ for a single disorder instance with $K=20$ cycles in ${\hat{U}}_{{\rm{S}}}$. The square root of the autocorrelator, obtained by replacing ${\hat{U}}_{{\rm{F}}}^{t}$ with ${\hat{U}}_{{\rm{ECHO}}}$, ${A}_{\psi ,0}$, is also shown. Bottom panel: normalized autocorrelator, ${A}_{\psi }/{A}_{\psi ,0}$, as a function of *t*. **d**, Histograms of $|{A}_{\psi }|$ from a single disorder instance, averaged over cycles 30 and 31. Each histogram corresponds to a different number of scrambling cycles, $K$, and includes data from 500 random initial bit-string states before ${\hat{U}}_{{\rm{S}}}$.

Our measurement of $[\bar{A}]$ for 500 initial states in Fig. 3d provides clear evidence of initial-state independence. Still, a direct sampling of states is practically limited to small fractions of the computational basis (0.05% in this case) and would suffer from the exponential growth of the Hilbert space on larger systems. A more scalable alternative is to use random, highly entangled states to directly measure spectrally averaged quantities (quantum typicality^37–39^; see Supplementary Information). The autocorrelator $A$ averaged over all ${2}^{L}$ bit strings agrees, up to an error exponentially small in $L$, with ${A}_{\psi }=\langle \psi |\hat{Z}(0)\hat{Z}(t)|\psi \rangle$, where $|\psi$⟩ is a typical Haar-random many-body state in the Hilbert space of $L$ qubits. We prepare such a state by evolving a bit string with a random circuit ${\hat{U}}_{{\rm{S}}}$ of variable depth $K$ (Fig. 4b), and couple an ancilla qubit to the system to measure the two-time operator $\hat{Z}(0)\hat{Z}(t)$ (Fig. 4a). Experimental results for the error-mitigated, spectrally averaged signal ${A}_{\psi }/{A}_{\psi ,0}$ on qubit ${Q}_{11}$ (Fig. 4c) show behaviour consistent with a stable MBL-DTC. The effect of the state-preparation circuit ${\hat{U}}_{{\rm{S}}}$ is illustrated by the dependence of $\sigma$ for ${A}_{\psi }$ on $K$. As shown in Fig. 4d, $\sigma$ steadily decreases as $K$ increases, reducing from a value of 0.025 at $K=0$ to a value of 0.006 at $K=20$, while $\mu$ remains largely unchanged. This is consistent with the fact that $|\psi$⟩ becomes closer to a Haar-random state as $K$ increases. We use a single disorder instance to study the convergence of the quantum typicality protocol because disorder averaging independently leads to narrow distributions even for $K=0$ (Fig. 3b). Results for prethermal and thermalizing dynamics are shown in Supplementary Fig. 10.

The scaling with $L$ of the spectrally averaged autocorrelator, at a time $t\sim {\rm{poly}}(L)$, provides a sharp diagnostic: this saturates to a finite value in the MBL-DTC, while it scales to zero with increasing $L$ in the thermal phase and in prethermal cases where, for instance, a vanishing fraction of the spectrum of an appropriate ${\hat{H}}_{{\rm{eff}}}$ shows order (see Supplementary Information). While the averaged autocorrelator may be unduly affected by outlier states and/or long (but $O(1)$) thermalization times at small system sizes and times (thereby making the complementary bit-string analysis of Fig. 3 essential), the polynomial scaling of this protocol establishes a proof of principle for efficiently verifying the presence or absence of an MBL-DTC in a range of models as quantum processors scale up in size to surpass the limits of classical simulation^40^.

**Fig. 5:**
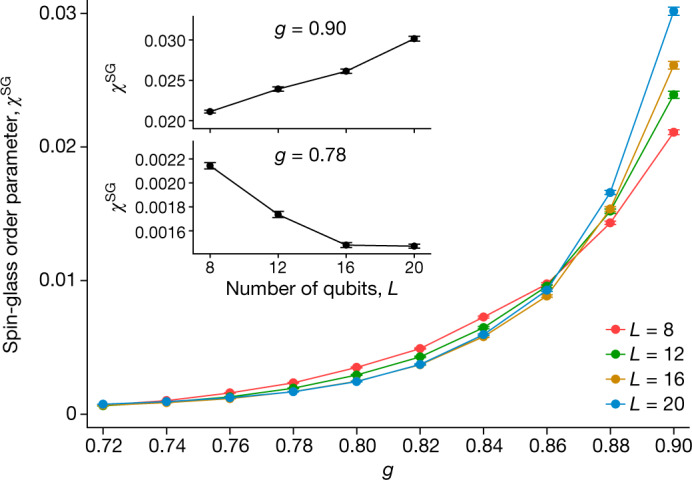
Estimating phase transition by varying system size. Disorder-averaged spin-glass order parameter ${\chi }^{{\rm{SG}}}$ as a function of $g$ for different chain lengths *L*, measured between $t=51$ and $t=60$. Error bars correspond to statistical errors alone and do not include hardware (for example, gate) errors. Inset shows the size dependence of ${\chi }^{{\rm{SG}}}$ for two different values of *g*. See Methods for measurement details.

Finally, we systematically vary $g$ in small increments and obtain an experimental finite-size analysis to establish the extent of the MBL phase and the transition out of it. Defining phases of matter, whether in or out of equilibrium, requires a limit of large system size. Thus, it is important to examine the stability of the MBL-DTC and thermalizing regimes observed in our finite-size quantum processor as the size of the system is increased. To address this, we measure an Edwards–Anderson spin-glass order parameter^41,42^

2 $${\chi }^{{\rm{SG}}}=\frac{1}{L-2}{\sum _{i\ne j}}^{\text{'}}{\langle {\hat{Z}}_{i}{\hat{Z}}_{j}\rangle }^{2}$$

(the primed sum excludes edge qubits ${Q}_{1}$, ${Q}_{L}$), as a function of time. This quantity measures the persistence of random (‘glassy’) spatial patterns in the initial bit-string state: at late times, *χ*^SG^ vanishes with increasing $L$ in the thermalizing phase $g < {g}_{{\rm{c}}}$, while it is extensive in the MBL-DTC $g > {g}_{{\rm{c}}}$. As a result, it is expected to show a finite-size crossing at $g\simeq {g}_{{\rm{c}}}$ (although the precise location is subject to strong finite-size and finite-time drifts^43,44^). Experimentally, ${\chi }^{{\rm{SG}}}$ is constructed from bit-string samples obtained by jointly reading out all qubits and then averaged over cycles and disorder instances (Fig. 5). The size of the qubit chain is varied by restricting the drive ${\hat{U}}_{{\rm{F}}}$ to contiguous subsets of 8, 12 and 16 qubits (as well as the entire 20-qubit chain). We observe increasing (decreasing) trends in ${\chi }^{{\rm{SG}}}$ versus $L$ when $g$ is above (below) a critical value ${g}_{{\rm{c}}}$. The data indicate $0.83\lesssim {g}_{{\rm{c}}}\lesssim 0.88$, consistent with numerical simulations (see Supplementary Information).

In conclusion, we have demonstrated the possibility of engineering and characterizing non-equilibrium phases of matter on a quantum processor, providing the experimental observation of an MBL-DTC. The scalability of our protocols sets a blueprint for future studies of non-equilibrium phases and phase transitions on complex quantum systems beyond classical simulability. The efficient verification of eigenstate order can inspire a general strategy for establishing whether a desired property, such as a particular phase, is in fact present in a quantum processor.

## Methods

### Edge qubits

In computing various site-averaged quantities such as $[\bar{A}]$ or ${\chi }^{{\rm{SG}}}$, we have excluded contributions from the edge qubits ${Q}_{1}$ and ${Q}_{20}$. This is because they may be affected by the presence of edge modes independent of the bulk DTC response^45^.

### Estimating distribution of autocorrelation functions

The measurements in Fig. 3a, b are conducted without error mitigation (that is, normalization via the echo circuits ${\hat{U}}_{{\rm{ECHO}}}$). This is primarily due to the already high number of experimental circuits that need to be measured given the large collection of initial states and disorder instances. Adding echo circuits to each of these instances would make the data acquisition time unfeasibly long. We note that the experimental conclusions for Fig. 3a, b are in agreement with noiseless simulation of the same circuit instances, which reveals the same features as experimental data. See Supplementary Fig. 9 for details.

### ‘Prethermalization’ in ${\hat{U}}_{{\rm{F}}}^{{\prime} }$ model

We refer to the circuit ${\hat{U}}_{{\rm{F}}}^{{\prime} }$ (with uniform angles ${\varphi }_{i}=-\,0.4$) used in Fig. 3 as prethermal. The choice of value for ${\varphi }_{i}$ is such that the dynamics is governed by an effective Hamiltonian ${\hat{H}}_{{\rm{eff}}}$ for long times (see Supplementary Information for a derivation). Strictly speaking, a prethermal DTC requires ${\hat{H}}_{{\rm{eff}}}$ to have a symmetry-breaking phase transition at a finite temperature ${T}_{{\rm{c}}}$—in that case, ordered initial states at temperatures $T < {T}_{{\rm{c}}}$ show long-lived oscillations (with an amplitude that depends on the equilibrium value of the symmetry-breaking order parameter at temperature *T* (ref. ^31^)). While short-ranged models in one dimension (such as the one under consideration) cannot have order at any finite temperature, thermal correlation lengths at low temperatures may still exceed the system size. This allows low-temperature states to show long-lived oscillations with a finite amplitude, even if the equilibrium order parameter is asymptotically zero for such states.

### Measurement of the spin-glass order parameter

In Fig. 5, every data point is averaged over 40 disorder instances and 10 cycles ($t=51$ to $t=60$). To construct ${\chi }^{{\rm{SG}}}$, we sample 40,000 bit strings at the output of ${\hat{U}}_{{\rm{F}}}^{t}$ for each cycle and disorder instance. To address the inhomogeneity of qubit coherence, smaller qubit chains are also averaged over different possible combinations of qubits. For example, $L=12$ is averaged over 12-qubit chains made from ${Q}_{1}$ to ${Q}_{12}$, ${Q}_{3}$ to ${Q}_{15}$ and so on. The $|0{\rangle }^{\otimes L}$state is used as the initial state for all disorder instances. Error bars are estimated by resampling data from the 40 disorder instances via the jackknife method.

### Comparison between many-body echo and single-qubit errors

The many-body echo circuits ${\hat{U}}_{{\rm{ECHO}}}={({\hat{U}}_{{\rm{F}}}^{\dagger })}^{t}{\hat{U}}_{{\rm{F}}}^{t}$ are chosen for characterizing decoherence effects since they capture the complex interplay between Floquet dynamics and single-qubit errors. More specifically, the decay of a particular observable (for example, $\langle \hat{Z}(t)\rangle$) depends not only on single-qubit error rates, but also on how much the quantum operator $\hat{Z}$ is ‘spread’ to different qubits over time. This effect is visible in the different decay rates for the echo data with $g=0.60$ and $g=0.97$ in Fig. 2. Nevertheless, for values of $g$ close to 1, the decay rate of local observables ${\bar{A}}_{0}$ should be close to single-qubit error rates^29^ and at least some basic comparison may be made.

A description of gate errors, characterized through cross-entropy benchmarking^40^, can be found in Supplementary Fig. 1. The single-qubit errors are also characterized through standard metrics of ${T}_{1}$, ${T}_{2}^{\ast }$ and ${T}_{2}^{{\rm{CPMG}}}$. We find *T*_1_ = 16.1 (5.3) µs across the 20-qubit chain, where the value in parenthesis represents the standard deviation. ${T}_{2}^{\ast }$, which is characterized through Ramsey measurements, is found to be ${T}_{2}^{\ast }=5.8$ (2.8) µs. ${T}_{2}^{{\rm{CPMG}}}$, characterized through CPMG measurements, is found to be ${T}_{2}^{{\rm{CPMG}}}=16.6$ (3.7) µs.

These values may be compared to the characteristic decay rates of the echo experiment (that is, ${\bar{A}}_{0}$ in Fig. 2c) at $g=0.97$, which are found to be 6.4 (1.1) µs across the qubit chain. Here the quantum system is strongly localized, and the decay of the echo experiment is dominated by single-qubit decoherence^29^. Given that this decay rate is closest to the value of ${T}_{2}^{\ast }$, the extrinsic decoherence in our experiments is probably limited by low-frequency noise (the main contributor to ${T}_{2}^{\ast }$) and, to some extent, energy relaxation and high-frequency noise as well. A more detailed characterization of decoherence mechanisms is left as the subject of future research.

### Classical computational complexity of DTC circuits

The computational complexity of DTC circuits in the thermal and critical regimes asymptotically scales as an exponent of depth and number of qubits. Even though our 20-qubit experiment can be simulated on classical computers, it demonstrates a scalable protocol that could be applied to larger systems with higher connectivity geometries beyond the capacity of classical algorithms. We expect the circuit- and geometry-dependent scaling exponent to be smaller than that for the two-dimensional random circuits implemented in refs. ^40,34^. Therefore, to challenge classical supercomputers, we would need DTC circuits larger than those in refs. ^40,34^. Calculation of this threshold is beyond the scope of this paper.

## Online content

Any methods, additional references, Nature Research reporting summaries, source data, extended data, supplementary information, acknowledgements, peer review information; details of author contributions and competing interests; and statements of data and code availability are available at 10.1038/s41586-021-04257-w.

## Supplementary information

Supplementary Information This file contains Sections I–VIII, including Figs. 1–10 and additional references.

## Data Availability

The experimental data contained in the main text and Supplementary Information are available for download at 10.5281/zenodo.5570676.

## Code availability

The Python simulation code used in theoretical analysis is available for download at 10.5281/zenodo.5570676.
