# Supplementary material for: Time-crystalline eigenstate order on a quantum processor
Source: Nature. 2021 Nov 30;601(7894):531–6. doi: 10.1038/s41586-021-04257-w (PMC8791837; doi:10.1038/s41586-021-04257-w)
Supplement: Supplementary file 1 — This file contains Sections I–VIII, including Figs. 1–10 and additional references. [file 41586_2021_4257_MOESM1_ESM.pdf]

nature portfolio

https://doi.org/10.1038/s41586-021-04257-w

Supplementary information

Time-crystalline eigenstate order on a quantum processor

In the format provided by the authors and unedited

Supplementary Information for "Time-Crystalline Eigenstate Order on a Quantum Processor"

Google AI Quantum and Collaborators

I. CPHASE GATE IMPLEMENTATION AND ERROR BENCHMARKING

An essential building block for the quantum circuits used to observe many-body localized DTC is the two-qubit gate $ZZ(\phi) = e^{-i\frac{\phi}{4}\hat{Z}_{\mathrm{a}}\hat{Z}_{\mathrm{b}}}$, which belongs to the more general family of Fermionic Simulation (FSIM) gates having the unitary form $\hat{U}_{\mathrm{FSIM}}$ [1]:

$$\begin{pmatrix} 1 & 0 & 0 & 0 \\ 0 & e^{i(\Delta_+ + \Delta_-)}\cos\theta & -ie^{i(\Delta_+ - \Delta_{-,\mathrm{off}})}\sin\theta & 0 \\ 0 & -ie^{i(\Delta_+ + \Delta_{-,\mathrm{off}})}\sin\theta & e^{i(\Delta_+ - \Delta_-)}\cos\theta & 0 \\ 0 & 0 & 0 & e^{i(2\Delta_+ - \phi)} \end{pmatrix}. \tag{S1}$$

Here θ is the two-qubit iSWAP angle and Δ_+, Δ_- and $\Delta_{-,\mathrm{off}}$ are phases that can be freely adjusted by single-qubit Z-rotations. In this parametrized representation, $ZZ(\phi) = \hat{U}_{\mathrm{FSIM}}(\theta = 0, \Delta_- = 0, \Delta_{-,\mathrm{off}} = 0, \phi = 2\Delta_+)$, which is equivalent to a CPHASE gate with conditional-phase ϕ and a single-qubit rotation $Z(\frac{\phi}{2})$ acting on each qubit. Precise single-qubit Z-control has already been demonstrated in our previous work [2]. Here, we focus on implementing CPHASE gates with a variable ϕ.

The qubits used in our experiment are superconducting transmon qubits with both tunable frequencies and tunable inter-qubit couplings. Due to the existence of higher states, a natural way to realize a CPHASE gate is to bring the $|11\rangle$ and $|02\rangle$ states of two coupled qubits close to resonance diabatically, allow the qubits to interact for a duration $\sim \frac{1}{\sqrt{8g^2+\epsilon^2}}$, before turning off the inter-qubit coupling and ramping the qubits back to their idle configurations. Here $|0\rangle$ and $|1\rangle$ are the ground and excited states of each qubit, $|2\rangle$ is a leakage state outside the computational space, g denotes the inter-qubit coupling (between the $|10\rangle$ and $|01\rangle$ states) and ϵ is the detuning between the $|11\rangle$ and $|02\rangle$ states during interaction. A schematic for the flux pulses to realize the CPHASE gate is shown in Fig. S1a.

Figure S1b shows simulated values of leakage, namely the probability of one qubit residing in $|2\rangle$ after the CPHASE gate, as a function of ϵ and maximum value of g during interaction, g_{max}. A narrow arc-like region, corresponding to a contour $8g_{\mathrm{max}}^2 + \epsilon^2 =$ constant, can be seen from the simulation. The values of g_{max} and ϕ along this contour are shown in the upper panel of Fig. S1c, where we see a full range of $\phi \in [-2\pi, 0]$ can be achieved for absolute detuning values of $|\epsilon|/2\pi < 100$ MHz. Since the $|01\rangle$ and $|10\rangle$ states are detuned by $\sim$200 MHz, their interaction remains dispersive during the CPHASE gate and therefore ensures a small iSWAP angle θ (we confirm this experimentally in the next section).

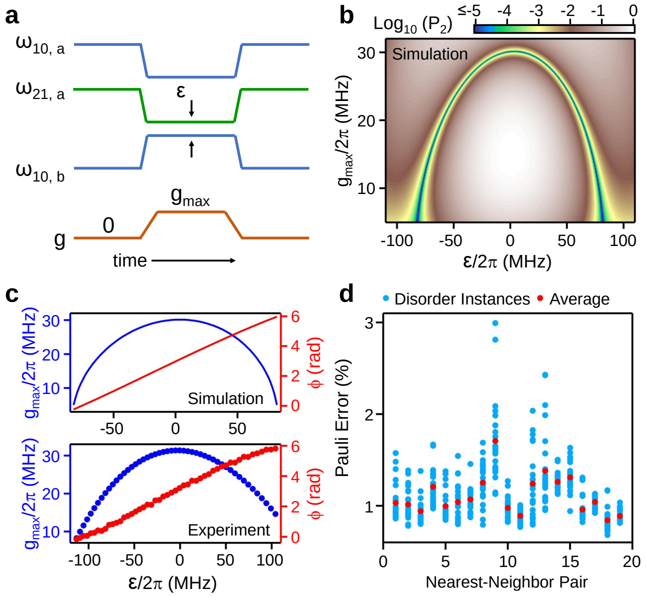

FIG. S1. **Implementing CPHASE gates with tunable transmon qubits. a,** Schematic of flux pulses used to realize a CPHASE gate. The frequencies of two coupled transmons, $\omega_{10,\,\mathrm{a}}$ and $\omega_{10,\,\mathrm{b}}$, are displaced from their idle positions into a configuration wherein $\omega_{10,\,\mathrm{b}}$ is detuned from $\omega_{21,\,\mathrm{a}}$ by an amount ϵ. At the same time, a flux pulse on the coupler turns on an inter-qubit coupling $g > 0$ with a maximum value of g_{max} for a fixed duration of $\sim$18 ns. **b,** Simulated values of leakage, P_2, as a function of ϵ and g_{max}, using typical device parameters and pulse shapes. **c,** The values of g_{max} and conditional-phase ϕ at the locations of minimum leakage, plotted for different values of ϵ. Upper panel shows simulated results and lower panel shows representative experimental values obtained from one pair of qubits. **d,** Pauli error rates for each neighboring pair of qubits in the 20-qubit chain used by the experiment, obtained from parallel XEB. Each error rate includes contributions from two random single-qubit gates ($\pi/2$ rotations around a random axis along the equatorial plane of the Bloch sphere) and a CPHASE gate. Data are shown for 24 disorder instances, with each instance including a different random set of ϕ_i across the qubit chain. Red dots show the average error of each qubit pair.

Experimentally, the leakage-minimizing value of g_{max} is detected for a discrete set of ϵ via $|2\rangle$ state readout and the corresponding ϕ is then calibrated using unitary tomography [3]. A polynomial fit is then performed to infer values of ϵ and g_{max} for intermediate values of ϕ, thereby achieving a continuous family of CPHASE gates with fully tunable ϕ. Example experimental calibration data for ϵ, g_{max} and ϕ are included in the bottom panel

of Fig. S1c. Excellent agreement with numerical results is found. The discrepancy in values of ϵ likely arises from deviation between the actual pulse shapes on the quantum processor and those used in the numerical simulation.

To estimate the errors of typical CPHASE gates, we perform cross-entropy benchmarking (XEB) similar to previous works [1, 2]. Here the gates are characterized in parallel and therefore include errors arising from any cross-talk effects. The XEB results for 24 random combinations of ϕ_i across the 20-qubit chain used in the main text are shown in Fig S1d, where we have used the so-called "cycle" Pauli error as the metric. A cycle Pauli error includes errors from two single-qubit gates and a single CPHASE gate. In computing the XEB fidelities, we have also assumed the CPHASE gate with the calibrated ϕ as the ideal gate [2]. As such, the Pauli errors include contributions from both incoherent effects such as qubit relaxation and dephasing, as well as coherent effects such as any mis-calibration in ϕ and residual values of θ. The error rates are observed to be relatively dependent on ϕ, a likely consequence of changes in coherent and incoherent errors when the pulse parameters are varied. Overall, we observe an average error rate of 0.011, comparable to gates used in our past works [1, 2].

II. FLOQUET CALIBRATION OF CPHASE GATES

After basic pulse-level calibration of CPHASE gates, the $ZZ(\phi)$ gate is then calibrated using the technique of Floquet calibration [4, 5]. Floquet calibration utilizes periodic circuits which selectively amplify different parameters within $\hat{U}_{\mathrm{FSIM}}$, allowing for sensitive detection and rectification of small coherent errors for such quantum gates. Our past works have primarily focused on calibrating the iSWAP-like family of gates, where $\theta \gg \phi$. For ZZ gates, the opposite limit $\phi \gg \theta$ is true and the optimal calibration circuits are in some cases different from our previous works. In this section, we present the gate sequences and example calibration results for the ZZ gates. For a detailed description of the underlying theory of Floquet calibration, the reader is directed to our previous publications [4, 5].

A. Calibration of Δ_+, Δ_- and ϕ

The calibration circuits for Δ_- are illustrated in Fig. S2a and comprise two Ramsey-like measurements: Each qubit is separately initialized along the x-axis of the Bloch sphere, $|X\rangle$. A total number of d FSIM gates are then applied, which in general rotate the excited qubit around the z-axis of the Bloch sphere due to non-zero single-qubit phases within the uncalibrated $\hat{U}_{\mathrm{FSIM}}$. At the end of the sequence, a Z gate with a rotation angle ξ is applied to the excited qubit, followed by a $\sqrt{Y}$ gate. The resulting $|1\rangle$ state population, P_1, is then measured. Example data for P_1 of each qubit are shown in the bottom panel of Fig. S2a, which are fitted to a cosine function $P_1 = B_0 + B_1 \cos(\xi + \xi_0)$ where B_0, B_1 and ξ_0 are fitting parameters. The value of Δ_- is then equal to $\frac{\xi_a - \xi_b}{2d}$, where ξ_a (ξ_b) is the fitted ξ_0 for Q_{a} (Q_{b}). This equivalence may be understood through the fact that $2\Delta_-$ is the difference in the degree of local Z ro-

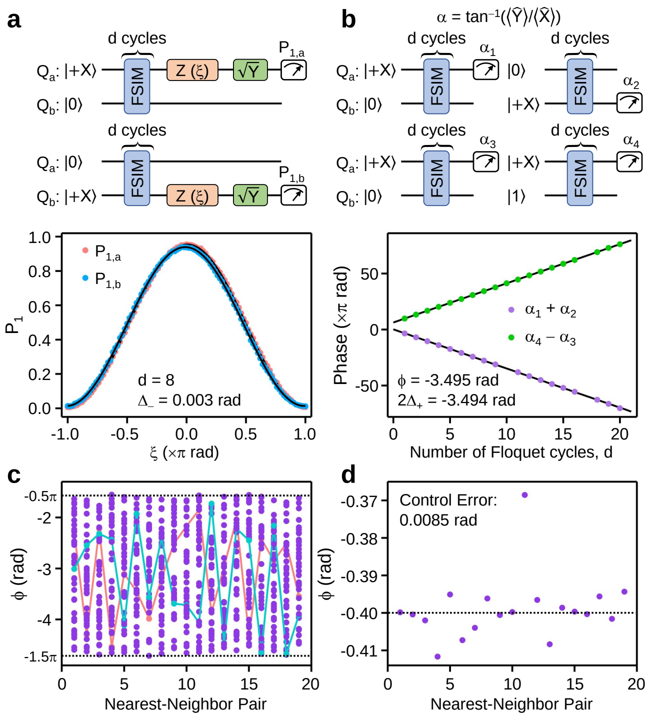

FIG. S2. **Floquet calibration of single-qubit and conditional phases for CPHASE-like gates. a,** Top panel: Gate sequences for calibrating the Δ_- angle of the FSIM. Bottom panel: Example experimental data for $P_{1,\mathrm{a}}$ and $P_{1,\mathrm{b}}$ ($|1\rangle$ state population for qubits Q_{a} and Q_{b}, respectively) as functions of ξ. The circuit depth is fixed at $d = 8$. Solid black lines show fits to a cosine function for each qubit, which allow Δ_- to be extracted. **b,** Top panel: Gate sequences for calibrating Δ_+ and ϕ. For each of the 4 gate sequences, the $\langle\hat{X}\rangle$ and $\langle\hat{Y}\rangle$ projections of the Bloch vector for a given qubit are measured at the end, from which an angle $\alpha = \tan^{-1}\left(\langle\hat{Y}\rangle / \langle\hat{X}\rangle\right)$ is computed. Bottom panel: Example experimentally obtained phase sums ($\alpha_1 + \alpha_2$) and differences (α_4 - α_3) as functions of d, number of cycles in the Floquet gate sequences. Solid black lines show linear fits, the slopes of which determine ϕ and Δ_+. **c,** Experimentally measured ϕ for each neighboring pair of qubits in the 20-qubit chain. Results from 40 disorder instances are plotted. The blue and red connected dots indicate the values of two particular instances, while all other instances are shown as disconnected purple dots. Dashed lines corresponding to $\phi = -0.5\pi$ and $\phi = -1.5\pi$. **d,** Experimental measurements of ϕ when a target value is set at -0.4 (dashed line) for all nearest-neighbor pairs. An average deviation of 0.0085 rad is found between the target and measured values of ϕ.

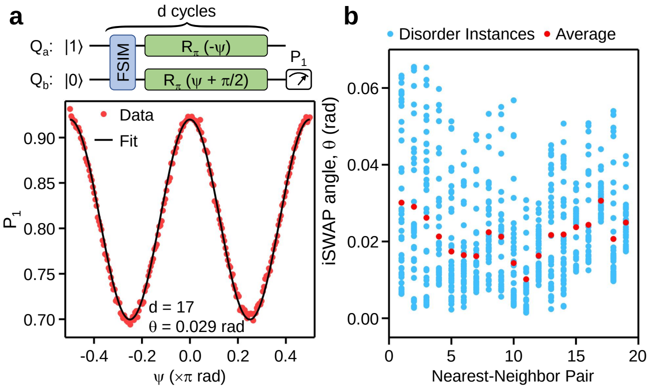

FIG. S3. **Floquet calibration of small iSWAP angles. a,** Top: Periodic circuit for calibrating θ: Each cycle includes an FSIM gate, followed by two single-qubit rotations $R_\pi(-\psi)$ and $R_\pi(\psi+\pi/2)$. After d cycles, the excited state population P_1 of Q_b is measured. Bottom: Example experimental data at a fixed depth $d=17$, showing P_1 as a function of ψ. Solid black line shows fit to a sinusoidal function, the amplitude of which determines the value of θ. **b,** Experimentally measured θ for each neighboring pair of qubits in the 20-qubit chain. Results from 40 disorder instances are plotted as blue dots, and the average value for each qubit pair is plotted as red dots. Overall, θ has a mean value of 0.022 rad and a standard deviation 0.014 across all qubit pairs and disorder instances.

tations undergone by each qubit after the application of one FSIM gate between them.

The phases Δ_+ and ϕ are calibrated using four periodic circuits sharing a similar structure, as indicated in the top panel of Fig. S2b. For Δ_+, we again separately prepare each qubit in the $|X\rangle$ state while leaving the other qubit in $|0\rangle$. The FSIM gate is then applied d times. At the end of the sequence, two tomographic measurements are performed on the excited qubit to determine the angle of its Bloch vector, $\alpha = \tan^{-1}\left(\langle\hat{Y}\rangle / \langle\hat{X}\rangle\right)$. The total accumulated phase $\alpha_1+\alpha_2$, where α_1 (α_2) is α for Q_a (Q_b), is equivalent to $2d\Delta_+$. This equivalence arises from the fact that $2\Delta_+$ physically corresponds to the sum of the degrees of local Z rotations on both qubits after the application of one FSIM gate.

The conditional-phase ϕ is calibrated by preparing one qubit (Q_a) in $|X\rangle$ and comparing α when the other qubit (Q_b) is initialized in either the $|0\rangle$ or the $|1\rangle$ state. A nonzero ϕ will cause the two angles, α_3 and α_4, to differ by an amount $\alpha_4-\alpha_3=-d\phi$ [3]. Example experimental values of $\alpha_1+\alpha_2$ and $\alpha_4-\alpha_3$ as a function of d are shown in the bottom panel of Fig. S2b. The slopes of both data sets allow Δ_+ and ϕ to be extracted, which are seen to be very close to the target condition $2\Delta_+=\phi$. Figure S2c shows experimentally calibrated values of ϕ across the 20-qubit chain, for a total of 40 disorder instances. It can be seen that ϕ falls within the intended range of $[-0.5\pi, -1.5\pi]$. Figure S2d shows the calibrated values of ϕ when each ϕ_i has a target value of -0.4. Comparing the measured values of ϕ with the target value, we find a small control error of 0.0085 rad for ϕ.

B. Calibration of θ

For iSWAP-like gates within the FSIM family, the iSWAP angle θ can be accurately calibrated by setting $\Delta_-=0$ and applying FSIM gates in succession, which leads to population transfer between the two qubits (initialized in $|10\rangle$) with a period of $\frac{\pi}{\theta}$. Discrete fourier transform of qubit populations therefore allow θ to be determined with very high precision [5]. However, CPHASE-like gates typically have small iSWAP angles and such a technique is no longer as effective, since the period for one population transfer can be very long and the calibration data are complicated by noise effects.

To circumvent such a problem, we have designed a new gate sequence for calibrating small values of θ: Let us consider the composite unitary $\hat{U}_\mathrm{COM}=\hat{U}_\mathrm{FSIM}X_\mathrm{a}Y_\mathrm{b}$ where X_a and Y_b are X and Y π-rotations of Q_a and Q_b, respectively. $\hat{U}_\mathrm{COM}$ has the following matrix form:

$$\begin{pmatrix} 0 & 0 & 0 & -i \\ 0 & -e^{-i\Delta_{-,\mathrm{off}}}\sin\theta & i\cos\theta & 0 \\ 0 & -i\cos\theta & e^{i\Delta_{-,\mathrm{off}}}\sin\theta & 0 \\ ie^{-i\phi} & 0 & 0 & 0 \end{pmatrix}. \quad \text{(S2)}$$

Here we have set Δ_- and Δ_+ to 0 for simplicity (nonzero values of these phases will not affect the calibration scheme discussed below). With simple algebra, it can be seen that for qubits initialized in the $|10\rangle$ state, the excited state population of Q_b after applying $\hat{U}_\mathrm{COM}$ d times (d being odd) is bounded by two values: For $\Delta_{-,\mathrm{off}}=0$, $P_1=\cos^2\theta\approx 1$ for small values of θ. For $\Delta_{-,\mathrm{off}}=\frac{\pi}{2}$, $P_1=\cos^2(d\theta)$. The difference between these two values is $\cos^2\theta-\cos^2(d\theta)$ which is approximately $\sin^2(d\theta)$ for $\theta\approx 0$. As such, varying $\Delta_{-,\mathrm{off}}$ and measuring the peak-to-peak amplitude of P_1 allows determination of θ. Compared to the fourier-transform technique, the method here requires relatively short circuit depth, as a iSWAP angle of 0.02 rad would yield a peak-to-peak amplitude of 0.1 in P_1 for $d=17$, which can be resolved with a relatively small number of single-shot measurements.

The experimental Floquet circuit for calibrating θ is shown in the upper panel of Fig. S3a. Here, we have injected a variational angle ψ into the single-qubit π-rotations. Varying ψ effectively changes χ of the FSIM gate. The experimental calibration data for a given pair of qubits are shown in the bottom panel of Fig. S3a, where we see oscillations of P_1 as a function of ψ. Fitting the data to a sinusoidal function allows a peak-to-peak amplitude of 0.22 to be determined, which corresponds to a iSWAP angle of $\theta=0.029$ rad.

The iSWAP angles for all qubit pairs of the 20-qubit chain are shown in Fig. S3b, where we have included data for 40 disorder instances in ϕ. A small average value of 0.022 rad is found for the qubit chain, with the fluctuation between disorder instances understood as a result

of different detunings between the $|01\rangle$ and $|10\rangle$ states during the flux pulses of different gates.

III. DERIVATION OF EFFECTIVE HAMILTONIANS

The bit-string energies shown in the inset to Fig. 3b are based on effective Hamiltonians $\hat{H}_{\rm eff}$ that, in certain limits, approximate the effect of the unitary circuit over two periods, $\hat{U}_F^2 \approx e^{-2i\hat{H}_{\rm eff}}$. Here we derive the relevant $\hat{H}_{\rm eff}$ operators for the models in Fig. 3b.

A. Uniform $\phi_i = -0.4$

For the model with uniform CPHASE angles $\phi_i \equiv \bar{\phi} = -0.4$ and random single-qubit Z rotation angles $h_i \in [-\pi, \pi]$, a period of the time evolution is represented by

$$\hat{U}'_F = \hat{U}_z[\bar{\phi}, \mathbf{h}]\hat{U}_x[\pi - 2\epsilon] \tag{S3}$$

with

$$\begin{aligned} \hat{U}_x[\theta] &= e^{-i\frac{\theta}{2}\sum_n \hat{X}_n} , \\ \hat{U}_z[\bar{\phi}, \mathbf{h}] &= e^{-i\sum_n (\bar{\phi}/4)\hat{Z}_n\hat{Z}_{n+1} + (h_n/2)\hat{Z}_n} . \end{aligned}$$

We have also defined the detuning $\epsilon = \frac{\pi}{2}(1-g)$; in the following we take $\epsilon \ll 1$, i.e. g close to 1. The evolution over two periods is given by

$$\begin{aligned} (\hat{U}'_F)^2 &= \hat{U}_z[\bar{\phi}, \mathbf{h}]\hat{U}_x[\pi - 2\epsilon]\hat{U}_z[\bar{\phi}, \mathbf{h}]\hat{U}_x[\pi - 2\epsilon] \\ &= \hat{U}_z[\bar{\phi}, \mathbf{h}]\hat{U}_x[-2\epsilon]\hat{U}_z[\bar{\phi}, -\mathbf{h}]\hat{U}_x[-2\epsilon] \end{aligned} \tag{S4}$$

where we have used the comutation properties of the perfect π pulse $\hat{U}_x[\pi] = \prod_n \hat{X}_n$. Next, we note that $\hat{U}_z[\bar{\phi}, -\mathbf{h}] = \hat{U}_z[\bar{\phi}, 0]\hat{U}_z[0, \mathbf{h}]^\dagger$; acting by conjugation with $\hat{U}_z[0, \mathbf{h}]$ on $\hat{U}_x[-2\epsilon]$ gives

$$(\hat{U}'_F)^2 = \hat{U}_z[\bar{\phi}, 0]e^{i\epsilon\sum_n \cos(h_n)\hat{X}_n + \sin(h_n)\hat{Y}_n}\hat{U}_z[\bar{\phi}, 0]\hat{U}_x[-2\epsilon] . \tag{S5}$$

The effective Hamiltonian $\hat{H}_{\rm eff}$, satisfying $(\hat{U}'_F)^2 \approx e^{-2i\hat{H}_{\rm eff}}$, is then given to leading order in ϵ, $|\bar{\phi}/4| \ll 1$ via the Baker-Campbell-Hausdorff (BCH) formula:

$$\begin{aligned} \hat{H}_{\rm eff} = \sum_n \frac{\epsilon}{2}[(1 + \cos(h_n))\hat{X}_n + \sin(h_n)\hat{Y}_n] + \\ + \frac{\bar{\phi}}{4}\hat{Z}_n\hat{Z}_{n+1} . \end{aligned} \tag{S6}$$

Thus, for any bit-string $\mathbf{s} \in \{0, 1\}^L$, the energy of the associated computational basis state $|\mathbf{s}\rangle = |s_1\rangle |s_2\rangle \cdots |s_L\rangle$ is

$$E_{\mathbf{s}} = \langle \mathbf{s}| \hat{H}_{\rm eff} |\mathbf{s}\rangle = \frac{\bar{\phi}}{4}\sum_n (-1)^{s_n + s_{n+1}} , \tag{S7}$$

which simply counts the number of "domain walls" (i.e. bonds where $s_i \neq s_{i+1}$) in $\mathbf{s}$. Thus the polarized and staggered bit-strings (having 0 and $L-1$ "domain walls", respectively) lie near the edges of the spectrum in all realizations.

We note that, strictly speaking, a prethermal DTC requires $\hat{H}_{\rm eff}$ to have a symmetry breaking transition at a finite critical temperature T_c. In this case, ordered initial states at temperatures $T < T_c$ show long-lived oscillations with an amplitude that depends on the equilibrium value of the (symmetry breaking) order parameter at temperature T [6]. As short-ranged models in one dimension (such as the one under consideration) cannot have order at finite temperature, this model is not prethermal in the sense we just described. However, thermal correlation lengths may still exceed the size of the system when the latter is small enough. This allows low-temperature states of $\hat{H}_{\rm eff}$ to show long-lived oscillations with a finite amplitude, even if the equilibrium order parameter is asymptotically zero for such states.

B. Disordered $\phi_i \in [-1.5\pi, -0.5\pi]$

In the MBL DTC drive $\hat{U}_F$ we set the average CPHASE angle to $\bar{\phi} = -\pi$, which (being ~ 10 times larger than in the previous case) breaks the final step in the derivation of Eq. (S6). We can however use another approach, valid if $\phi_i = -\pi + \delta\phi_i$, with $|\delta\phi_i|$ sufficiently small. In Eq. (S5) we replace $\bar{\phi}$ by $\pi + \boldsymbol{\delta\phi}$, and noting that $\hat{U}_z[\pi, 0] = \hat{U}_z[-\pi, 0]$ [7] we have

$$\begin{aligned} \hat{U}_F^2 = \hat{U}_z[\boldsymbol{\delta\phi}, 0]e^{i\epsilon\sum_n(\cos(h_n)\hat{Y}_n - \sin(h_n)\hat{X}_n)(\hat{Z}_{n-1} + \hat{Z}_{n+1})} \\ \times \hat{U}_z[\boldsymbol{\delta\phi}, 0]\hat{U}_x[-2\epsilon] . \end{aligned} \tag{S8}$$

If ϵ, $|\delta\phi_i| \ll 1$, leading-order BCH yields

$$\begin{aligned} \hat{H}_{\rm eff} = \sum_n \frac{\epsilon}{2}[\hat{X}_n + \cos(h_n)\hat{Y}_n(\hat{Z}_{n-1} + \hat{Z}_{n+1})] \\ + \frac{\delta\phi_n}{4}\hat{Z}_n\hat{Z}_{n+1} - \frac{\epsilon}{2}\sin(h_n)\hat{X}_n(\hat{Z}_{n-1} + \hat{Z}_{n+1}) \end{aligned} \tag{S9}$$

The energy of a bit-string state $|\mathbf{s}\rangle$ is

$$E_{\mathbf{s}} = \langle \mathbf{s}| H_F^{(0)} |\mathbf{s}\rangle = \sum_n \frac{\delta\phi_n}{4}(-1)^{s_n + s_{n+1}} . \tag{S10}$$

Unlike the result in Eq. (S7), this does not single out special bit-strings. More specifically, under disorder averaging all bit-strings have the same energy: $\overline{E_{\mathbf{s}}} = 0$.

In our model, the $|\delta\phi_i|$ angles are not small (they vary in $[-0.5\pi, 0.5\pi]$) so all orders in BCH should be included for an accurate result – the above is only meant to be a qualitative approximation. Nevertheless, the independence of (disorder-averaged) quantities from the choice of bit-string can be proven exactly for this model.

All bit-string states are obtained as $|\mathbf{s}\rangle = \hat{X}_{\mathbf{s}} |\mathbf{0}\rangle$, where $|\mathbf{0}\rangle = |00\ldots00\rangle$ is the polarized state and $\hat{X}_{\mathbf{s}} =$

$\prod_{i:s_i=1} \hat{X}_i$ flips the qubits where $s_i = 1$. We will show that all bit-string states give rise to the same dynamics as the polarized one, up to a change in the realization of disorder. Indeed, the change of basis that maps $|\mathbf{s}\rangle$ to $|\mathbf{0}\rangle$ acts on $\hat{U}_F$ as

$$\hat{X}_{\mathbf{s}} \hat{U}_F \hat{X}_{\mathbf{s}} = \hat{U}_z[\boldsymbol{\phi}', \mathbf{h}'] \hat{U}_x[\pi g] \tag{S11}$$

where $\phi'_i = (-1)^{s_i+s_{i+1}} \phi_i$ and $h'_i = (-1)^{s_i} h_i$. $\boldsymbol{\phi}'$ and $\mathbf{h}'$ almost define another valid realization of disorder, except that wherever $s_i \neq s_{i+1}$, we have $\phi'_i \in [0.5\pi, 1.5\pi]$ (as opposed to $\phi_i \in [-1.5\pi, -0.5\pi]$). This can be remedied by setting $\phi''_i = \phi'_i - 2\pi \in [-1.5\pi, -0.5\pi]$, and noting that $e^{-i\frac{\pi}{2}\hat{Z}_i\hat{Z}_{i+1}} \propto e^{-i\frac{\pi}{2}\hat{Z}_i} e^{-i\frac{\pi}{2}\hat{Z}_{i+1}}$, so that the excess 2π CPHASE angle can be transferred to single-qubit rotations: $\hat{U}_z[\boldsymbol{\phi}', \mathbf{h}'] \propto \hat{U}_z[\boldsymbol{\phi}'', \mathbf{h}'']$, where $h''_i = h'_i$ if $s_{i-1} = s_{i+1}$, or $h'_i + \pi$ otherwise. Thus the dynamics of bit-string $|\mathbf{s}\rangle$ under disorder realization $(\boldsymbol{\phi}, \mathbf{h})$ is mapped to the dynamics of $|\mathbf{0}\rangle$ under a different realization $(\boldsymbol{\phi}'', \mathbf{h}'')$. Further, the mapping between realizations conserves the (uniform) measure over the intervals $\phi_i \in [-1.5\pi, -0.5\pi]$, $h_i \in [-\pi, \pi]$. Thus after averaging over disorder, all bit-strings are equivalent to each other.

IV. ECHO CIRCUIT FOR NOISE MITIGATION

The "echo" circuit $\hat{U}_{\text{ECHO}}$ used to define the normalization A_0 in Fig. 2c and Fig. 4d consists of t steps of forward time evolution under $\hat{U}_F$ and t steps for "backward" time evolution under $\hat{U}_F^\dagger$. In the absence of noise, the two cancel exactly. Thus deviations from this outcome quantify the impact of noise.

A. Circuit inversion

Our device allows the calibration of a continuous family of CPHASE angles on each bond and their use during a single circuit run. Thus it is possible to concatenate the forward and backward time evolutions $\hat{U}_F^t$ and $(\hat{U}_F^\dagger)^t$ directly. However, the two-qubit gates in $\hat{U}_F^\dagger$ would have in general different fidelity than those in $\hat{U}_F$. As a result, the decoherence during $\hat{U}_{\text{ECHO}}$ would differ from that during $\hat{U}_F$.

To circumvent this, we effectively invert the circuit $\hat{U}_F$ without changing the two-qubit gates, thus keeping the fidelity unchanged during the backward time evolution. The key idea is to apply X rotations on even qubits, $\hat{P}_{\pi,e} \equiv \prod_{n=1}^{L/2} \hat{X}_{2n}$, before and after each period of the circuit that is to be inverted. Indeed conjugating $\hat{U}_F$ by $\hat{P}_{\pi,e}$ changes the sign of all ϕ_n CPHASE angles. It also changes the sign of single-qubit Z rotation angles h_n on even sites. The sign of remaining h_n fields and of g, as well as the relative order of the X and Z parts of the drive, can be changed at the level of single-qubit gates, with minor effects on fidelity.

In practice, after t cycles of $\hat{U}_F$, we apply the single-qubit rotations $\hat{P}_{\pi,e}$ only once, and then switch to a unitary $\hat{V}_F$ which has the same 2-qubit gates as $\hat{U}_F$ but different single-qubit gates chosen so that $\hat{P}_{\pi,e} \hat{V}_F \hat{P}_{\pi,e} = \hat{U}_F^\dagger$ (as explained above). Finally we measure in the computational basis and flip the logical value of all bits at even positions (this is equivalent to acting with $\hat{P}_{\pi,e}$ at the final time but avoids any fidelity losses). This way, we manage to effectively invert the circuit without altering two-qubit gate fidelities.

B. Measuring the effect of decoherence

Let us model noise as a single-qubit channel

$$\mathcal{E}_p = \left(1 - \frac{4p}{3}\right) \mathcal{I} + \frac{4p}{3} \mathcal{D} \tag{S12}$$

where $\mathcal{I}$ is the identity channel ($\mathcal{I}(\hat{\rho}) = \hat{\rho}$), $\mathcal{D}$ is the single-qubit erasure channel ($\mathcal{D}(\hat{\rho}) \propto I$), and p denotes the error rate. We assume the noise acts symmetrically before and after each iteration of the unitary circuit (while realistically noise acts *during* the entire evolution, this is a good approximation as long as $p \ll 1$). Then the time evolution of the system over a period is described by a quantum channel

$$\hat{\rho} \mapsto \mathcal{E}_{p/2}^{\otimes L} \circ \mathcal{U}_F \circ \mathcal{E}_{p/2}^{\otimes L}(\hat{\rho}) \equiv \Phi(\hat{\rho}) \tag{S13}$$

where $\mathcal{U}_F(\hat{\rho}) = \hat{U}_F \hat{\rho} \hat{U}_F^\dagger$. Similarly the inverted time evolution is given by

$$\hat{\rho} \mapsto \mathcal{E}_{p/2}^{\otimes L} \circ \mathcal{U}_F^\dagger \circ \mathcal{E}_{p/2}^{\otimes L}(\hat{\rho}) \equiv \Phi^\dagger(\hat{\rho}) \tag{S14}$$

where $\mathcal{U}_F^\dagger(\hat{\rho}) = \hat{U}_F^\dagger \hat{\rho} \hat{U}_F$, and the last equality holds because $\mathcal{E}_p$ is self-adjoint. The entire echo circuit sequence is thus described by the channel $(\Phi^\dagger)^t \circ \Phi^t$. The expectation value of $\hat{Z}_i$ after the circuit is given by

$$\langle \hat{Z}_i \rangle_{\mathbf{s}}^{\text{echo}} \equiv \text{Tr}\left(\hat{Z}_i (\Phi^\dagger)^t \circ \Phi^t(|\mathbf{s}\rangle\langle\mathbf{s}|)\right) \tag{S15}$$

The temporal autocorrelator between $\hat{Z}_i$ before and after the echo circuit is simply $(-1)^{s_i} \langle \hat{Z}_i \rangle_{\mathbf{s}}^{\text{echo}}$. Averaging over all bit-strings yields

$$\begin{aligned} A_0^2 &\equiv \frac{1}{2^L} \sum_{\mathbf{s}} (-1)^{s_i} \langle \hat{Z}_i \rangle_{\mathbf{s}}^{\text{echo}} \\ &= \frac{1}{2^L} \sum_{\mathbf{s}} \text{Tr}\left[\hat{Z}_i (\Phi^\dagger)^t \circ \Phi^t(\hat{Z}_i |\mathbf{s}\rangle\langle\mathbf{s}|)\right] \\ &= \frac{1}{2^L} \text{Tr}\left[(\Phi^t(\hat{Z}_i))^2\right] = \|\Phi^t(\hat{Z}_i)\|^2 / \|\hat{Z}_i\|^2 \, , \end{aligned} \tag{S16}$$

where we have used the definition of adjoint channel, $(\hat{V}, \Phi(\hat{W})) = (\Phi^\dagger(\hat{V}), \hat{W})$, relative to the trace inner product $(\hat{V}, \hat{W}) = \text{Tr}(\hat{V}^\dagger \hat{W})$. Thus from the protocol outlined above one extracts the decay of operator norm

$\|\hat{Z}_i(t)\| \sim A_0 \|\hat{Z}_i(0)\|$ which is the leading effect of decoherence. The ratio A/A_0 in Fig. 2d thus gives the overlap between *normalized* operators,

$$\frac{A}{A_0} = \left(\frac{\hat{Z}_i(0)}{\|\hat{Z}_i(0)\|} \middle| \frac{\hat{Z}_i(t)}{\|\hat{Z}_i(t)\|} \right) . \quad \text{(S17)}$$

To conclude, we remark on the behavior of $A_0 \propto \|\hat{Z}_i(t)\|$ in the different phases. Under depolarizing noise, a Pauli string $\hat{P}$ is damped by a factor $e^{-\gamma w(\hat{P})}$, where γ is fixed by the Pauli error rate and $w(\hat{P})$ is the *weight* of the Pauli string $\hat{P}$, i.e. the number of qubits on which $\hat{P} \neq \hat{I}$. Thus spatially extended operators decay more quickly than single-qubit ones. Deep in the MBL phase, the local integrals of motion ("l-bits") are exponentially localized with some localization length $\xi = O(1)$, giving an average Pauli weight $1 < \bar{w} < \xi$. Thus $\|\hat{Z}_i(t)\| \sim e^{-\gamma \bar{w} t}$, where $\bar{w}$ effectively modulates the decay rate. In the thermal phase, local operators spread ballistically, so that $\bar{w} \propto t$. This causes a faster-than-exponential decay at early times, as seen in Fig. 2c of the main text. Then once the operator has spread to the whole size of the system, the decay becomes exponential again.

V. SPECTRAL AVERAGES VIA QUANTUM TYPICALITY

Quantum typicality[8–10] states that, for any observable $\hat{O}$ in a Hilbert space of dimension 2^L, the expectation value $\langle \hat{O} \rangle_\psi$ on a *random state* ψ sampled from the unitarily invariant (Haar) measure obeys these statistical properties:

$$\mathbb{E}_\psi \langle \hat{O} \rangle_\psi = \langle \hat{O} \rangle_\infty \quad \text{(S18)}$$

$$\mathrm{Var}_\psi \langle \hat{O} \rangle_\psi = \frac{1}{2^L + 1} \left(\langle \hat{O}^2 \rangle_\infty - \langle \hat{O} \rangle_\infty^2 \right) \quad \text{(S19)}$$

where $\langle \hat{O} \rangle_\infty \equiv 2^{-L} \mathrm{Tr}(\hat{O})$ denotes the expectation value on the infinite-temperature state. Thus for large L, the matrix element $\langle \hat{O} \rangle_\psi$ is distributed as a Gaussian centered at the infinite-temperature value with an extremely narrow standard deviation, $\simeq 2^{-L/2}$, which enables the measurement of spectrally-averaged quantities with exponentially high accuracy from a single pure state.

A. Scrambling circuit and approach to typicality

In the main text we report data on spectrally-averaged autocorrelators $\langle \hat{Z}_i(0) \hat{Z}_i(t) \rangle_\infty$ obtained with a method based on the idea above, i.e by evaluating $\langle \hat{Z}_i(0) \hat{Z}_i(t) \rangle_\psi$ on a state $|\psi\rangle$ which is close to typical random states in the Hilbert space. In order to prepare a random state $|\psi\rangle$, we start with a bit-string state and evolve it via a scrambling circuit $\hat{U}_S$, as also proposed in Ref. [11]. This consists of K layers of CZ gates (CPHASE with angle

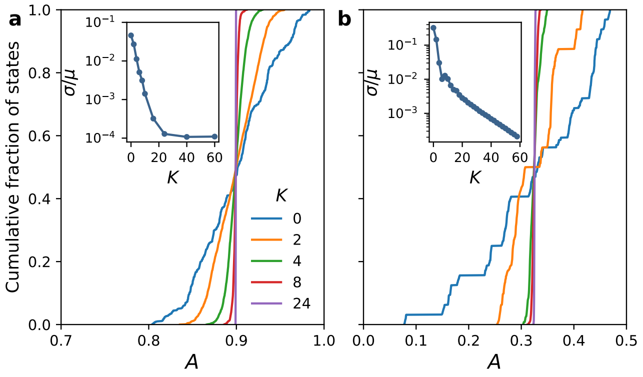

FIG. S4. **Simulation of quantum typicality protocol.** **a**, Cumulative distribution of autocorrelators A from a set of 2000 bit-string states pre-processed by a depth-K random circuit $\hat{U}_S$ as described in the text, for variable K. We set $g = 0.94$ (MBL DTC phase). The realization of disorder is fixed and A is computed at time $t = 30$ on qubit Q_{11} in a chain of $L = 20$ qubits. Inset: relative standard deviation σ/μ decreases exponentially in K and approaches the random-state variance ($< 2^{-L/2}$) after depth $K \simeq L$. **b**, Same plot for noisy simulations (depolarizing noise, error rate $p = 0.5\%$ per 2-qubit gate, exact density matrix simulations) of qubit Q_7 in a chain of $L = 12$ qubits, where we include all 4096 bit-string states. Inset: relative standard deviation σ/μ. σ decreases indefinitely with K due to decoherence, while μ is not affected.

$\phi = \pi$) and random single-qubit gates (rotations around a random direction on the XY plane by an angle θ sampled uniformly in $[0.4\pi, 0.6\pi]$). The single-qubit gates vary randomly in both space and time, so that $\hat{U}_S$ is not a Floquet circuit. After a number of layers $K = O(L)$ (we neglect decoherence for now), the prepared state $|\psi\rangle = \hat{U}_S |\mathbf{s}\rangle$ behaves like typical random vectors in the Hilbert space, so that $\langle \psi | \hat{O} | \psi \rangle = \langle \hat{O} \rangle_\infty + \delta$, where the error δ (a random variable dependent on the choice of bit-string $\mathbf{s}$ and of scrambling circuit $\hat{U}_S$) has zero mean and variance $\sim 2^{-\min(L, cK)}$ for some constant $c > 0$, i.e., the variance shrinks with increasing K as the state becomes gradually more random, until it saturates to the quantum typicality result Eq. (S19). In Fig. S4a we show the results of exact numerical simulations that confirm this picture. For this family of random circuits, we find $c \simeq 0.36$ (from a fit to the data in the inset to Fig. S4a).

B. Ancilla protocol

To measure two-time correlators $\langle \psi | \hat{Z}_i(0) \hat{Z}_i(t) | \psi \rangle$ in the "pseudorandom states" $|\psi\rangle$ defined above, we use an interferometric protocol similar to the one employed in Ref. [2]. We introduce an ancilla qubit initialized in state $|+X\rangle = (|0\rangle + |1\rangle)/\sqrt{2}$ alongside the system qubits Q_1, $\ldots Q_L$ which are initialized in a bit-string state $|\mathbf{s}\rangle$. We evolve the system qubits with the scrambling circuit $\hat{U}_S$

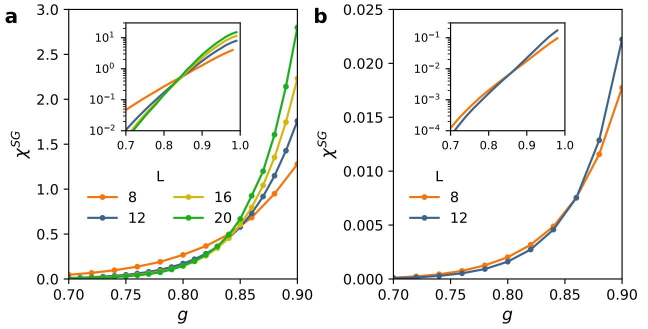

FIG. S5. **Numerical simulations of spin glass order parameter.** **a**, Ideal (noiseless) dynamics. χ^{SG} averaged over even times between $t = 50$ and $t = 60$, over initial bit-string states and over realizations of disorder. At least 4000 realizations are averaged at sizes $L = 8$, 12, and 16, at least 300 at $L = 20$. Inset: same data on a semilogarithmic scale. **b**, Noisy dynamics (exact density matrix simulations). Noise is modeled by single-qubit depolarizing channels with Pauli error rate $p = 0.8\%$ after each 2-qubit gate. χ^{SG} is averaged over even times between $t = 50$ and $t = 60$, over initial bit-string states, and over realizations of disorder. 1000 realizations are used at $L = 8$, 200 at $L = 12$. Inset: same data on a semilogarithmic scale.

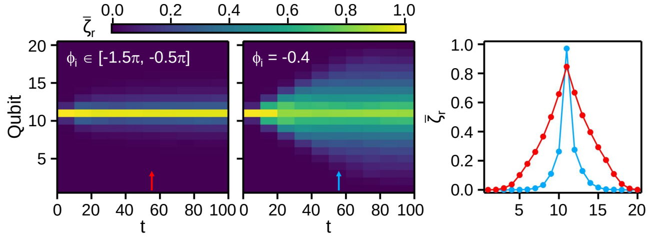

FIG. S6. **Numerical simulations of correlation measurements.** Noiseless simulation of the experiment in Fig. 3d of the main text. Here we simulate the fractional change in $\langle \hat{Z}(t) \rangle$, $\overline{\zeta}_r$ (see definition in main text), due to a single bit-flip at Q_{11} in initial condition. The simulation is averaged over 1000 disorder instances for both $\phi_i \in [-1.5\pi, -0.5\pi]$ and $\phi_i = -0.4$.

for depth K, obtaining a joint state $|\psi\rangle_{\text{sys}} |{+X}\rangle_{\text{a}}$. Then we apply a CZ gate between the ancilla and system qubit i, so that the state "branches" into the superposition

$$\frac{1}{\sqrt{2}} \left(|\psi\rangle_{\text{sys}} |0\rangle_{\text{a}} + \hat{Z}_i |\psi\rangle_{\text{sys}} |1\rangle_{\text{a}} \right) \tag{S20}$$

We then evolve the system under the Floquet drive $\hat{U}_F$ for t periods and again apply a CZ between the ancilla and system qubit i, which gives

$$\frac{1}{\sqrt{2}} \left(\hat{U}_F^t |\psi\rangle_{\text{sys}} |0\rangle_{\text{a}} + \hat{Z}_i \hat{U}_F^t \hat{Z}_i |\psi\rangle_{\text{sys}} |1\rangle_{\text{a}} \right) \tag{S21}$$

Finally, we measure the ancilla in the X basis. The expectation value of the measurement is

$$\begin{aligned} \langle \hat{X}_{\text{a}} \rangle &= \frac{1}{2} \langle \psi | \hat{U}_F^{-t} \hat{Z}_i \hat{U}_F^t \hat{Z}_i |\psi\rangle + \text{c.c.} \\ &= \text{Re} \langle \psi | \hat{Z}_i(t) \hat{Z}_i(0) |\psi\rangle \\ &\simeq \langle \hat{Z}_i(0) \hat{Z}_i(t) \rangle_\infty \end{aligned} \tag{S22}$$

where the last line follows from quantum typicality if $|\psi\rangle$ is random. On a sufficiently large system, and for large enough K (number of scrambling cycles), this protocol gives the spectrum-averaged temporal autocorrelator from a single measurement.

C. Effect of noise during the scrambling circuit

The above discussion neglects decoherence and treats the states during the protocol as pure. Since K must grow with L for the protocol to succeed, it is especially important to understand whether noise during the random state preparation process has a negative impact on the result.

One can repeat the previous discussion with quantum channels instead of unitary operators: the system starts in pure state $|\mathbf{s}\rangle\langle\mathbf{s}|_{\text{sys}} |{+X}\rangle\langle{+X}|_{\text{a}}$ and evolves under the scrambling dynamics into $\hat{\rho}_{\text{sys}} |{+X}\rangle\langle{+X}|_{\text{a}}$, where $\hat{\rho} = \Phi_S(|\mathbf{s}\rangle\langle\mathbf{s}|)$ and Φ_S is a quantum channel representing the noisy version of the scrambling circuit $\hat{U}_S$ (we neglect decoherence on the ancilla qubit). The protocol then proceeds analogously to the noiseless case and yields the final state

$$\begin{aligned} \hat{\rho}'_{\text{sys,a}} = \frac{1}{2} \big[& \Phi^t(\hat{\rho})_{\text{sys}} |0\rangle\langle 0|_{\text{a}} + (\hat{Z}_i \Phi^t(\hat{Z}_i \hat{\rho}))_{\text{sys}} |1\rangle\langle 0|_{\text{a}} \\ & + (\Phi^t(\hat{\rho}\hat{Z}_i)\hat{Z}_i)_{\text{sys}} |0\rangle\langle 1|_{\text{a}} \\ & + (\hat{Z}_i \Phi^t(\hat{Z}_i \hat{\rho} \hat{Z}_i)\hat{Z}_i)_{\text{sys}} |1\rangle\langle 1|_{\text{a}} \big] \end{aligned} \tag{S23}$$

where Φ is the noisy version of the Floquet evolution $\hat{U}_F$. The expectation of $\hat{X}_{\text{a}}$ on this state is

$$\begin{aligned} \langle \hat{X}_{\text{a}} \rangle &= \frac{1}{2} \text{Tr} \left[\hat{Z}_i \Phi^t(\hat{Z}_i \hat{\rho}) + \Phi^t(\hat{\rho}\hat{Z}_i)\hat{Z}_i \right] \\ &= \frac{1}{2} \text{Tr}[\{\hat{Z}_i(t), \hat{Z}_i(0)\}\hat{\rho}] \end{aligned} \tag{S24}$$

where we have defined $\hat{Z}_i(t) = (\Phi^\dagger)^t[\hat{Z}_i]$ as the Heisenberg-picture evolution of $\hat{Z}_i$ with noise.

To see that Eq. (S24) approximates the infinite-temperature expectation value $\langle \hat{Z}_i(0)\hat{Z}_i(t) \rangle_\infty$, we observe that under a random unitary circuit, noise can be approximated by a global depolarizing channel[1]: $\Phi_S(|\mathbf{s}\rangle\langle\mathbf{s}|) \approx f^K \hat{U}_S |\mathbf{s}\rangle\langle\mathbf{s}| \hat{U}_S^\dagger + (1 - f^K)\hat{I}/2^L$, i.e. a sum of the ideal evolution under $\hat{U}_S$ and the fully mixed state $\hat{I}/2^L$ ($\hat{I}$ is the identity matrix), parametrized by a fidelity $f < 1$. However both the ideal scrambled state $\hat{U}_S |\mathbf{s}\rangle$ and the fully-mixed state $\hat{I}/2^L$ accurately reproduce the infinite-temperature expectation value. Thus decoherence during the random state preparation process may in fact

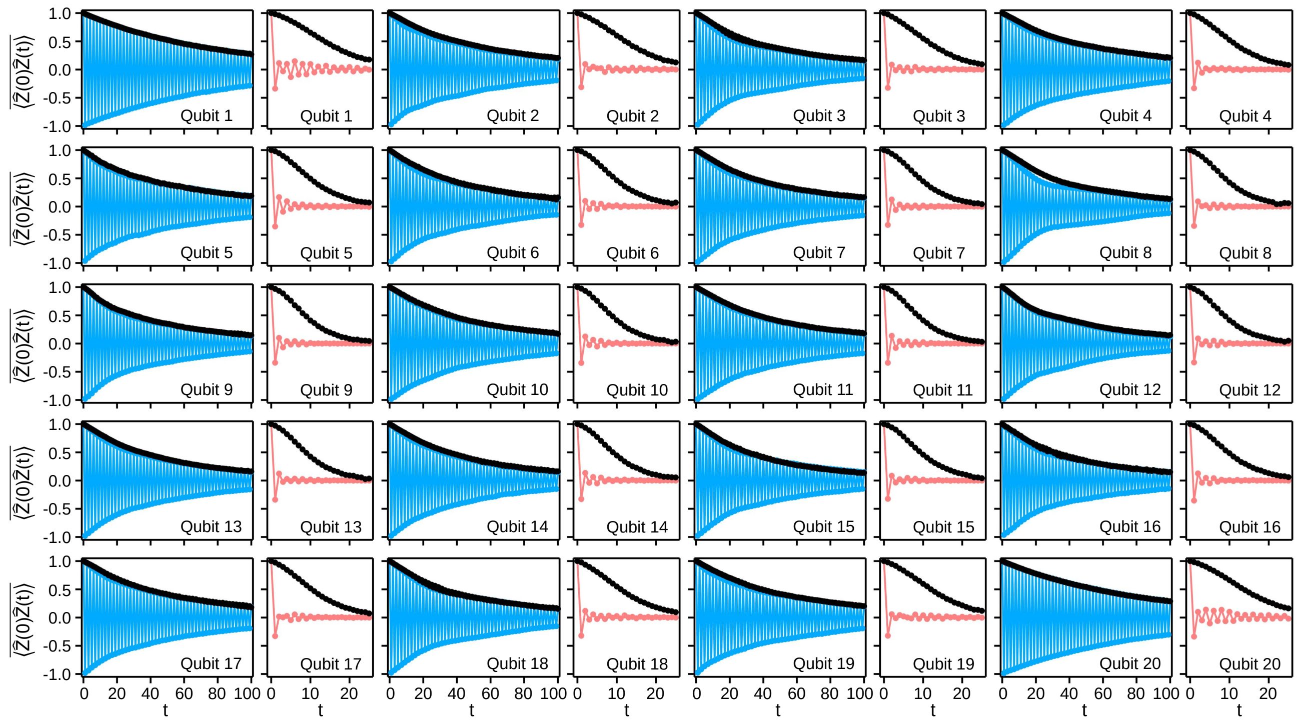

FIG. S7. **Autocorrelators for all qubits.** Experimentally obtained autocorrelators $\overline{A} = \overline{\langle \hat{Z}(0)\hat{Z}(t)\rangle}$, shown for all qubits in the system. The parameters are $\phi_i \in [-1.5\pi, -0.5\pi]$, $h_i \in [-\pi, \pi]$ and $g = 0.97$ (blue) and 0.60 (red). Data is averaged over 36 instances of random disorder and initial states, same as Fig. 2c of the main text. Black symbols represent results of the echo experiment, i.e. $\overline{A_0} = \sqrt{\langle \hat{Z}\hat{U}^\dagger_{\mathrm{ECHO}}\hat{Z}\hat{U}_{\mathrm{ECHO}}\rangle}$.

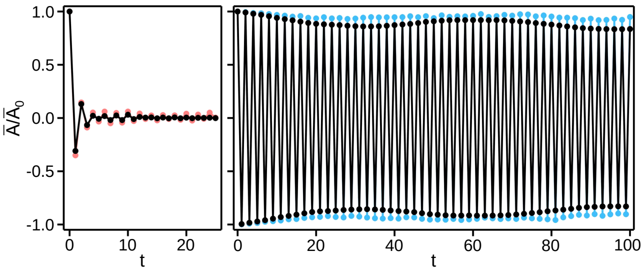

FIG. S8. **Comparison between error-mitigated autocorrelators and noise-free simulations.** Left: Error-mitigated aucorrelator $\overline{A}/\overline{A_0}$ (red), reproduced from the qubit 11 experimental data in Fig. 2d of the main text, and noise-free simulation results using the same disorder and initial state instances (black). $g = 0.60$ here. Right: Same as left but with $g = 0.97$ and experimental data represented by blue symbols.

be helpful, rather than harmful (as long as the circuit $\hat{U}_S$ is temporally random, so as to avoid any nontrivial steady states). This is confirmed by the results of exact density matrix simulations of $L = 12$ qubits in the presence of depolarizing noise, in Fig. S4b. The variance between bit-string states falls exponentially in K (depth of $\hat{U}_S$) even below the ideal quantum typicality limit of Eq. (S19). The subsequent decay is purely due to decoherence: the scrambled state $\Phi_S(\hat{\rho})$ asymptotically approaches the fully mixed state $\hat{I}/2^L$ as the noisy circuit is made deeper.

VI. NUMERICAL RESULTS ON SPIN GLASS ORDER PARAMETER

Here we show results of numerical simulations of the spin glass order parameter used to perform a finite-size analysis of the phases in the main text. We define the order parameter as

$$\chi^{SG} = \frac{1}{L-2}\sum_{i\neq j}{}' \langle \hat{Z}_i\hat{Z}_j\rangle^2 \qquad \text{(S25)}$$

where the primed sum excludes the edges (qubits Q_1 and Q_L) in order to remove the effects of edge modes from bulk physics. In a phase with glassy order all the expectation values $\langle \hat{Z}_i\hat{Z}_j\rangle$ are finite and χ^{SG} is extensive ($\sim L$). Otherwise, all expectation values asymptotically vanish and $\chi^{SG}/L \to 0$.

In Fig. S5a we show results of numerical simulations of χ^{SG} in the absence of noise, at times t between 50 and 60 cycles, as the length of the qubit chain is scaled from $L = 8$ to $L = 20$. A finite-size crossing is visible near

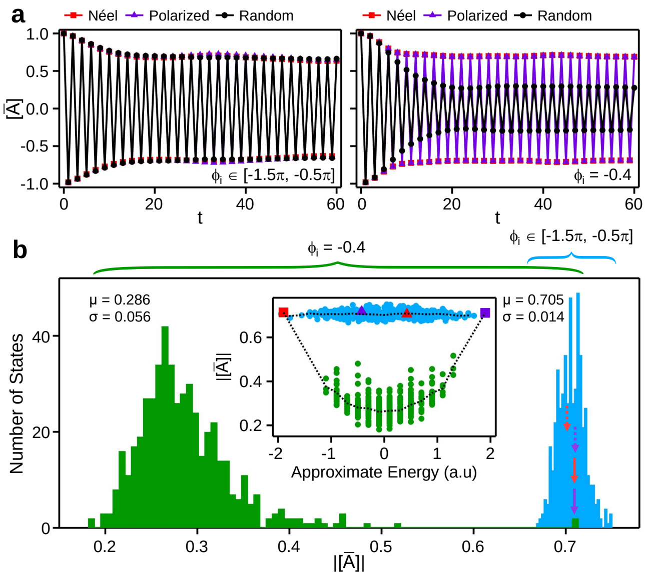

FIG. S9. **Initial state dependence from noiseless simulation.** **a**, Noiseless numerical simulation of the disorder- and position-averaged autocorrelators $[\overline{A}]$ as functions of t. The initial states, value of g and disorder instances are identical to the experimental plots in Fig. 3a of the main text. **b**, Noiseless numerical simulation of $|[\overline{A}]|$ averaged over cycles 30 and 31 and the same disorder instances as in panel a. The same 500 random initial states as used in Fig. 3b of the main text are used in producing the histograms. Purple (red) arrow indicates locations of the polarized (Néel) states, for the uniform-coupling $\phi_i = -0.4$ (solid arrows) and disordered-coupling $\phi_i \in [-1.5\pi, -0.5\pi]$ (dashed arrows) cases. Inset: same collection of $|[\overline{A}]|$ plotted over the energies of the bit-string states, calculated from the effective Hamiltonian $\hat{H}_{\text{eff}}$ approximating the drive (see main text). Dashed lines show averaged values within energy windows separated by 0.2.

$g_c \simeq 0.83$, separating a side of parameter space (at larger g) where χ^{SG} grows with L, indicative of the MBL-DTC phase, from one (at smaller g) where χ^{SG} decreases with L, indicative of a thermalizing phase. We also note that the finite-size crossing in these data slowly drifts towards higher g as t increases (not shown), as expected from slow thermalization on the ergodic side near the transition.

Repeating the same analysis in the presence of noise yields the data in Fig. S5b. We model noise as a single-qubit depolarizing channel with Pauli error rate $p = 0.8\%$ acting on both qubits after each 2-qubit gate. Simulations are carried out by exact evolution of the density matrix, which is memory-intensive and limits the available system sizes to $L \leq 12$ within reasonable computational resources. (We use this method rather than quantum trajectories[12] because the latter method is impractical for this calculation: as χ^{SG} is a nonlinear function of the state, each expectation $\langle \hat{Z}_i \hat{Z}_j \rangle$ must be averaged over trajectories separately for each disorder realization). We find that, as a result of noise, the finite-size crossing drifts to a slightly higher value of $g_c \simeq 0.86$, consistent

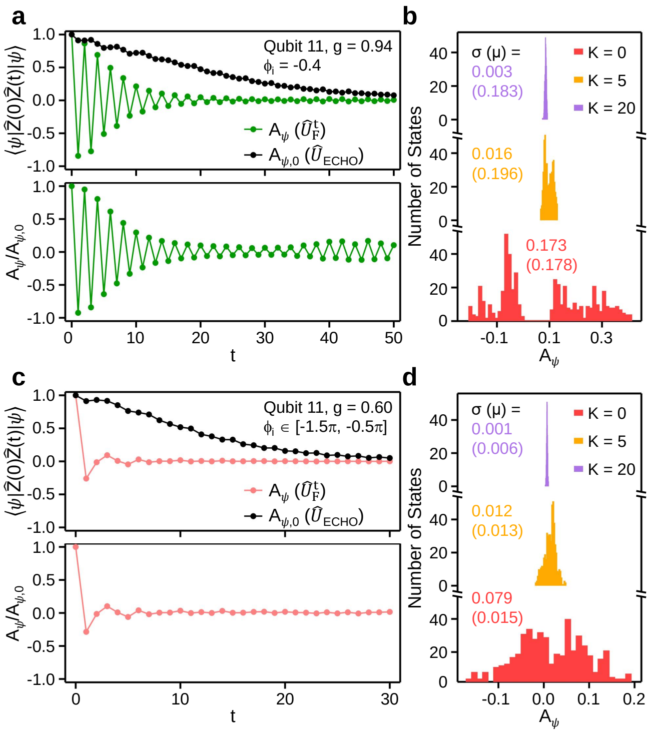

FIG. S10. **Average spectral response of prethermal and thermal dynamics.** **a, c**, Upper panels: A_ψ for a single disorder instance with $K = 20$ cycles in $\hat{U}_{\text{S}}$. The square-root of the autocorrelator obtained by replacing $\hat{U}_{\text{F}}^t$ with $\hat{U}_{\text{ECHO}}$, $A_{\psi,0}$, is also shown. Bottom panels: Normalized autocorrelator, $A_\psi / A_{\psi,0}$, as a function of t. Panel a corresponds to an instance of prethermal dynamics ($\phi_i = -0.4$ and $g = 0.94$) whereas panel c is an instance of thermalizing dynamics ($\phi_i \in [-1.5\pi, -0.5\pi]$ and $g = 0.60$). **b, d**, Histograms of A_ψ from a single disorder instance taken at $t = 12$ (panel b, prethermal dynamics) and $t = 4$ (panel d, thermalizing dynamics). Each histogram corresponds to a different number of scrambling cycles, K, and includes data from 500 random initial bit-string states fed through the scrambling circuit. See Fig. 4 of the main text for details of the protocol.

with the experimental estimate of the phase transition point $0.84 \lesssim g_c \lesssim 0.88$. We note that the noisy simulations in Fig. S5b do not include the effects of read-out error and that the depolarizing noise model is not guaranteed to be a quantitatively accurate approximation in these structured circuits. Even so, the simulated data is in good quantitative agreement with the experimental data for the selected value of the error rate p—for example we see that at $L = 12$ and $g = 0.90$, both Fig. S5b and Fig. 5 in the main text give $\chi^{SG} \simeq 0.02$.

VII. NUMERICAL RESULTS OF CORRELATION MEASUREMENTS

The noiseless simulation of the experiment in Fig. 3d of the main text is shown in Fig. S6.

Here we generate a total of 1000 disorder instances for both $\phi_i \in [-1.5\pi, -0.5\pi]$ and $\phi_i = -0.4$. The values of $\langle \hat{Z}(t) \rangle$ are simulated with the two initial conditions $|00000000000000000000\rangle$ (ζ_1) and $|00000000001000000000\rangle$ (ζ_2), and the ratio $\overline{\zeta}_\mathrm{r}$ is computed using the same method as the main text.

It is seen that the ratio $\overline{\zeta}_\mathrm{r}$ from the noise simulation is quite similar to the experimentally measured values, despite no active error-mitigation for this particular quantity. This is likely attributed to the fact that decoherence introduces a damping factor that is approximately the same for both the nominator ($|\overline{\zeta_1 - \zeta_2}|$) and denominator ($|\zeta_1| + |\zeta_2|$) used to compute $\overline{\zeta}_\mathrm{r}$. Consequently, their effects are canceled out after dividing the two quantities.

VIII. ADDITIONAL EXPERIMENTAL DATA AND NUMERICAL SIMULATION

A. Autocorrelators and Comparisons with Noise-Free Dynamics

Figure S7 shows experimentally obtained autocorrelators $\overline{A}$ for all qubits, along with results from the echo circuits.

Figure S8 shows the error-mitigated autocorrelators $\overline{A}/\overline{A}_0$ from Fig. 2d of the main text and noise-free simulation results using the same disorder and initial state instances.

B. Noiseless Simulation of Initial State Dependence

Figure S9 shows the simulated values of the disorder- and position-averaged autocorrelators $|\overline{A}|$ using the same disorder and initial state instances as Fig. 3a and Fig. 3b of the main text. Similar to the experimental data, we see a lower mean (μ) and larger relative standard deviation (σ/μ) for the "prethermal" quantum circuits with $\phi = -0.4$ compared to the MBL-DTC circuits with $\phi \in [-1.5\pi, -0.5\pi]$. In particular, the outlier states (e.g. Néel and polarized states) are clearly visible in the prethermal circuits and absent in the MBL-DTC circuits. The energy dependence is also clearly visible in the prethermal circuits whereas it is absent in the MBL-DTC circuits. These observations are consistent with the experimental observations in Fig. 3a and Fig. 3b, even without the need for error-mitigation (i.e. normalization via results of echo circuits).

C. Spectrally Averaged Response of Prethermal and Thermalizing dynamics

In Fig. 4 of the main text, we presented a protocol that leverages quantum typicality to probe the average response of the entire eigen-spectrum using the response from a single scrambled state. The protocol was demonstrated with MBL-DTC dynamics. Figure S10 shows similar data from a single instance of prethermal dynamics ($g = 0.94$ and $\phi_i = 0.4$) and a single instance of thermalizing dynamics ($g = 0.60$ and $\phi_i \in [-1.5\pi, -0.5\pi]$).

In both of these cases, we observe that the error-mitigated auto-correlators $A_\psi/A_{\psi,0}$ exhibits faster decay compared to the MBL-DTC data shown in Fig. 4 of the main text. Furthermore, in each case, we also observe that the distribution of A_ψ becomes considerably narrower as the number of scrambling cycles K is increased from 0 to 20, consistent with the expectation from quantum typicality.

[1] Arute, F. *et al.* Quantum supremacy using a programmable superconducting processor. *Nature* **574**, 505–510 (2019).

[2] Mi, X. *et al.* Information scrambling in quantum circuits. *Science* eabg5029 (2021).

[3] Foxen, B. *et al.* Demonstrating a continuous set of two-qubit gates for near-term quantum algorithms. *Phys. Rev. Lett.* **125**, 120504 (2020).

[4] Arute, F. *et al.* Observation of separated dynamics of charge and spin in the fermi-hubbard model. *Preprint at https://arxiv.org/abs/2010.07965* (2020).

[5] Neill, C. *et al.* Accurately computing the electronic properties of a quantum ring. *Nature* **594**, 508–512 (2021).

[6] Else, D., Bauer, B. & Nayak, C. Prethermal phases of matter protected by time-translation symmetry. *Phys. Rev. X* **7**, 011026 (2017).

[7] This is true up to single-qubit Z rotations on the edge qubits, if the chain has open boundary conditions. These could be cancelled by considering the evolution over 4 periods, with minor changes to the result (a cancellation of terms near the edges). For the sake of simplicity we will neglect this effect here.

[8] Popescu, S., Short, A. J. & Winter, A. Entanglement and the foundations of statistical mechanics. *Nat. Phys.* **2**, 754–758 (2006).

[9] Goldstein, S., Lebowitz, J. L., Tumulka, R. & Zanghì, N. Canonical typicality. *Phys. Rev. Lett.* **96**, 050403 (2006).

[10] Bartsch, C. & Gemmer, J. Dynamical typicality of quantum expectation values. *Phys. Rev. Lett.* **102**, 110403 (2009).

[11] Richter, J. & Pal, A. Simulating hydrodynamics on noisy intermediate-scale quantum devices with random circuits. *Phys. Rev. Lett.* **126**, 230501 (2021).

[12] Dalibard, J., Castin, Y. & Mølmer, K. Wave-function ap-

proach to dissipative processes in quantum optics. *Phys. Rev. Lett.* **68**, 580–583 (1992).
